# Characterizing NaV1.7 antisense analgesia through antagonist binding, mRNA expression, and neuraxial distribution

**DOI:** 10.1172/jci.insight.196154

**Published:** 2026-10-22

**Authors:** Kaue Malange, Julia Borges Paes Lemes, Briana Noble, Yuvicza Anchondo, Carlos E. Morado-Urbina, Saee Jadhav, Saurabh Yadav, Jingrui Ma, Vineet Augustine, Sara A. Dochnal, Chiraag Kambalimath, Marie Duong, Pedro Alvarez, Bethany Fitzsimmons, Curt Mazur, Holly B. Kordasiewicz, Kim Dore, Hien T. Zhao, Tony Yaksh

**Affiliations:** 1 Department of Anesthesiology, School of Medicine, UCSD, San Diego, California, USA.; 2 IONIS Pharmaceuticals, Inc., Carlsbad, California, USA.; 3 Department of Neurobiology, School of Biological Sciences, and; 4 Department of Neuroscience, School of Medicine, UCSD, San Diego, California, USA.

**Keywords:** Cell biology, Neuroscience, Ion channels, Pain, Pharmacology

## Abstract

Genome-targeting strategies to address NaV1.7-mediated signaling in nociceptive afferents are of great clinical interest given its high selectivity and persistent analgesic outcomes. In the present work, we analyze concentration-dependent effects of suppressing primary afferent NaV1.7 using the intrathecal delivery of an antisense oligonucleotide (ASO) on pain behaviors and demonstrate that NaV1.7 ASOs are likely to modulate pain traffic by blocking afferent signaling at the dorsal root ganglion (DRG) level. Intrathecal NaV1.7 ASOs induced a concentration-dependent gradient of knockdown in DRGs (lumbar to cervical) of *Scn9a* mRNA and ATTO488-ProTx-II binding in small DRG neurons, and a suppression of pain behaviors initiated by mechanical compression, inflammation, and the NaV1.7 agonist OD-1. The rostro-caudal reduction in mRNA correlated with ASO distribution, with implications for dose-related analgesic profiles. Marked reduction in DRG NaV1.7 binding was seen after intrathecal delivery of an ASO optimal dose, with high selectivity for small DRG neurons. Changes in sciatic nerve NaV1.7 binding were not detected, indicating a pain traffic modulation likely happening due to a block of afferent signaling at the DRG level.

## Abstract 2

The study enables a mechanistic understanding of how NaV 1.7 antisense therapeutics affect primary afferent trafficking and pain processing in rats.

## Introduction

Human observations and preclinical studies have validated the role of the voltage-gated sodium channel 1.7 (NaV1.7) as a target for pain control (1). Despite its promise as a target, there has been moderate success in creating small molecules and demonstrating efficacy in NaV1.7-triggered pain states (2, 3).

Collective evidence suggests that NaV1.7 expression occurs predominantly in C-polymodal nociceptors (4, 5), a conjecture that is widely accepted. Interestingly, recent evidence suggests that NaV1.7 mRNA and protein may also be present in second- and higher-order dorsal horn neurons that participate in nociceptive processing (6, 7).

Although NaV1.7 has a high degree of association with neuraxial nociceptive processing, its distribution has also been demonstrated in olfactory and gustatory processing systems (8), as well vagal afferent (9) and sympathetic neurons (10), raising concerns as to the potential effects of a systemically delivered drug on non–pain-related systems. This has raised the appreciation that specifically altering expression or function of NaV1.7 specifically in neuraxial nociceptive afferents has a particular virtue. Such anatomically selective knockout (KO) has been achieved using neuraxial delivery (11). Through this approach, small molecules, antisense oligonucleotides (ASOs), and various viral constructs have been shown to appear in dorsal root ganglion (DRG) neurons and to alter their transcriptional properties (12). The clinical utility of these platforms may be defined by their persistence (13).

The use of intrathecally delivered genome-modifying platforms, such as viral transfection, produces a long-lasting if not irreversible KO of protein expression, leading to persistent loss of function (12, 13). In contrast, intrathecal ASO therapeutics can in principle yield a conditional silencing and degradation of messenger ribonucleic acids (mRNAs), and long lasting, but reversible effect on protein synthesis (14–17).

In the present work, we addressed the concern that while quantification of mRNA provides an indirect index of channel expression, spatial or temporal disconnections related to protein synthesis, membrane transport, channel insertion, and internalization may prevent a 1 to 1 covariance (18, 19). To address this issue, we assessed the binding of the selective fluorophore-labeled NaV1.7 antagonist ATTO488–protoxin-II (hereafter, 488PTx-II) (20) to determine not only the amount of targeted binding at a specific site, but the fraction of cells expressing it. This approach allows us to estimate the effect on channel expression required to produce a given effect on pain behavior (12, 13, 21).

We found that ASOs delivered intrathecally are taken up by DRGs, spinal cord, and brain and selectively depress *Scn9a* mRNA synthesis, while the dose-dependent analgesia observed after intrathecal ASO covaries most closely with the dose-dependent changes in membrane NaV1.7 binding.

## Results

### Determining the optimal dose of ASO

#### Dose-response studies.

Spinal DRGs: The dose-response analysis revealed a gradient knockdown of *Scn9a* mRNA in DRGs through the spinal column. As the level leans towards rostral orientation, there is a shift to the right in the dose-response curve (Figure 1, A–C, displayed as percentage of *Scn9a* residual mRNA). Furthermore, the calculated IC_50_ changes significantly between the spinal levels, acquiring an approximately 10-fold difference between lumbar and cervical levels (Figure 1, A–C). For example, a 50% knockdown in DRG *Scn9a* mRNA can be achieved with 185 μg of ASO at the lumbar level, whereas for the cervical level this requires 1694 μg of ASO.

A plateau effect was seen for the 1000 and 3000 μg doses at the lumbar and thoracic DRGs, with a similar outcome detected at the cervical level. When compared with the mismatch ASO, these doses showed significant knockdown in DRGs across the 3 spinal levels (Supplemental Figure 1, A–C; supplemental material available online with this article; https://doi.org/10.1172/jci.insight.196154DS1). Accordingly, we defined 1000 μg as the minimum optimally effective ASO dose.

#### Spinal, trigeminal, and autonomic ganglia.

Previous work has shown that in addition to the spinal sensory ganglia expression, NaV1.7 has been observed in the trigeminal (22), nodose (23), and superior cervical ganglia (SCG) neurons (24), and recently in spinal neurons (6). We specifically assessed the effects of intrathecal NaV1.7 ASO at the optimally effective dose (1000 μg/10 μL). As expected, we observed in these animals a significant knockdown of *Scn9a* mRNA in lumbar DRGs and spinal cord, confirming the appropriate ASO delivery (Supplemental Figure 1, A, D, and H–K). We then observed in these animals a modest but statistically significant knockdown in *Scn9a* mRNA in trigeminal ganglia as well as the SCG (Supplemental Figure 1, E and F). In contrast, *Scn9a* mRNA in nodose ganglia was not affected by the lumbar intrathecal delivery of analgesic doses of NaV1.7 ASO, as compared with control- or missense-treated rats (Supplemental Figure 1G).

### ASO target selectivity, uptake, and toxicity

The intrathecal delivery of NaV1.7 ASO at the 1000 μg dose successfully led to a knockdown in NaV1.7 mRNA without affecting the other TTX-S (NaV1.1, -1.2, and -1.6) and TTX-R (NaV1.8 and -1.9) subunits (Figure 1D). This dose had effective uptake by cells at the spinal cord and DRG parenchyma (Supplemental Figure 1, H–K). In particular for DRGs, ASO particles could be detected in large and small neurons (Supplemental Figure 1, H and I; black and blue arrows, respectively). An extension of ASO signal could also be detected in the brain tissue (Supplemental Figure 2 for mismatch and Supplemental Figure 3 for NaV1.7 ASO).

No significant increase in glial activation markers *Aif-1* (IBA-1) and *Gfap* (GFAP) could be detected in DRGs, spinal cords, and brain cortex in comparison with PBS controls (Figure 1, E and F). These findings were aligned with the histopathological analysis, where mismatch and the NaV1.7 ASO optimal dose given intrathecally showed no significant indication of gliosis in the DRGs, spinal cord, and brain, when compared to PBS controls (Supplemental Figure 4, A–H for spinal cords and DRGs; and Supplemental Figure 5 for brain).

#### ASO targeting NaV1.7 dose-dependently affects nociceptive behavior evoked by intraplantar formalin.

After determining the ASO optimal dose, we tested its efficacy in different pain models. Initially, we performed a comparative study evaluating the same dose range used in the dose-response analysis (100–3000 μg in 10 μL) in the formalin model. As expected, the NaV1.7 ASO doses significantly knocked down *Scn9a* mRNA in DRGs and decreased formalin second-phase flinching in a different way. An effective analgesic profile was observed with the 1000 and 3000 μg doses (Figure 2A). Interestingly, despite the 50% knockdown of NaV1.7 mRNA seen at lower doses of ASOs (100 and 300 μg), no significant effect on formalin flinching could be detected (Figure 2, A and B).

#### ASOs targeting NaV1.7 decrease nociceptive behavior evoked by OD-1.

After investigating the effects of NaV1.7 knockdown in the formalin model, we evaluated the effects of the ASO optimal dose in modulating the intraplantar OD-1 flinching response induced by a NaV1.7 agonist (25). For this experiment, we selected the 1000 and 3000 μg doses of ASO, given their ability in providing significant *Scn9a* mRNA knockdown and affecting the nociceptive response in the formalin test. As shown in Figure 2, C–E, the nociceptive behavior evoked by the selective NaV1.7 agonist, OD-1, was significantly reduced by the intrathecal knockdown of *Scn9a* mRNA. Both ASO doses tested had similar outcomes in decreasing the flinching behavior triggered by intradermal injection of OD-1 (Figure 2C) with similar degrees in mRNA knockdown in the DRGs (Figure 2D) and spinal cord (Figure 2E).

#### ASO targeting NaV1.7 provides analgesia in male and female rats.

After a series of behavioral experiments to determine the effectiveness of the ASO optimal dose in formalin and OD-1 flinching, we investigated the analgesic activity of the optimal dose of NaV1.7 ASO in male and female rats. For this purpose, we evaluated the paw withdrawal thresholds for nociceptive response evoked by deep pressure through the Randall-Selitto test in male and female rats subjected to NaV1.7 knockdown. As shown in Figure 2, F–H, the selective knockdown of NaV1.7 in DRGs and spinal cords (Figure 2, G and H) markedly affected the mechanical nociception of NaV1.7 ASO–treated male and female rats that developed significant increases in paw withdrawal thresholds at 2 and 4 weeks after ASO intrathecal delivery. Mismatch and PBS controls had shown no effect (Figure 2F).

#### ASO targeting NaV1.7 provides analgesia in inflammatory pain model.

After we confirmed the analgesic effect from the NaV1.7 ASO in male and female rats, we investigated the ability of the ASO optimal dose to provide analgesia in a model where NaV1.7 blockers such as protoxin-II (26) would induce an analgesic profile, as in the carrageenan inflammatory pain model (27). As shown in Figure 2I, either NaV1.7 antagonist or ASO given intrathecally was able to reverse the tactile allodynia to baseline levels, as compared with controls, saline, or mismatch. The effects of NaV1.7 ASO were mirrored by an effective knockdown of *Scn9a* mRNA in the DRGs (Figure 2J). This analgesic profile evoked by both NaV1.7 antagonist and ASO in the same model highlights the covariance between transcriptional alterations and target functionality.

### Assessing the NaV1.7 binding phenotype in ASO animals

#### Characterization of 488PTx-II binding in the NaV1.7-expressing HEK cell platform.

We transfected NaV1.7-HaloTag channels in the HEK cell platforms used in this study (Supplemental Figure 6A, TMR-HaloTag signal). The HEK cells transfected with the NaV1.7-HaloTag plasmid exhibited significant fluorescence signals in comparison with untransfected controls (Supplemental Figure 6C). As previously mentioned, several concentrations of 488PTx-II were tested in our protocol to assess NaV1.7 binding in NaV1.7-transfected HEK cells. Only the 10 μM dose provided a significant signal in comparison with controls (Supplemental Figure 6, A–C). This fluorescence generated by the antagonist binding displayed strong covariance with the HaloTag fluorescence, indicating colocalization with the NaV1.7-expressing channels (Supplemental Figure 7, A–C). In our hands, 488PTx-II was able to generate a colocalized signal of 60% with the NaV1.7-HaloTag channels expressed (Supplemental Figure 7, D–G), as shown by the Pearson’s correlation coefficient (*r* = 0.6440 ± 0.0841) and in the Mander’s coefficient (0.6631 ± 0.0768).

#### 488PTx-II binding is reduced by Nav1.7 ASO in small DRG neurons.

After pharmacologically validating the NaV1.7 ASO and confirming the feasibility of the NaV1.7-binding approach in the HEK cell platform, we next conducted a series of experiments to elucidate the binding phenotype in animals treated with NaV1.7 ASO. Initially, our approach to label NaV1.7 channels was replicated in naive DRG cell cultures (Figure 3A). The DRG neurons labeled with 488PTx-II exhibited visible fluorescence signals in comparison with non-labeled controls (Figure 3A). Some neurons displayed punctate signal in the axon terminals and towards the cell body (Figure 3B, white arrows), which was confirmed through densitometry analysis (Figure 3C). The intrathecal NaV1.7 ASO led to a visible reduction in 488PTx-II signal in DRG cells in comparison with mismatch controls (Figure 3D). The quantitative fluorescence intensity for 488PTx-II in DRG cells of NaV1.7 ASO–treated animals was dramatically reduced in comparison with mismatch controls (Figure 3E). The majority percentage of cells expressing NaV1.7 binding for 488PTx-II were small-diameter neurons (Figure 3, F–H). This percentage was reduced in NaV1.7 ASO–treated animals, suggesting that the ASO treatment was able to knock down the NaV1.7 mRNA and lead to an absence of binding for the NaV1.7 antagonist, reflecting a lack of expression of the channel after NaV1.7 ASO (Figure 3F).

#### Reduced formalin flinching induced by NaV1.7 ASO covaries with decrease in 488PTx-II binding in DRGs but not at sciatic nerves.

The injection of formalin in mismatch animals led to a biphasic flinching response (Figure 4, A and B). This nociceptive behavior was significantly affected by the ASO optimal dose at second phase (1000 μg/10 μL; Figure 4, A and B). The lower ASO dose (300 μg/10 μL) had a profile similar to that of mismatch controls (Figure 4, A and B). In the whole-cell *Z*-stacks analyzed, the mismatch group displayed a significant increase in NaV1.7 binding at ipsilateral DRGs (Figure 4C) in comparison with contralateral DRG controls (Figure 4, C and D). This binding was significantly decreased in ipsilateral DRGs by the ASO optimal dose (Figure 4, C and D) but not by the lower dose tested (Figure 4, C and D). Interestingly, although different outcomes were observed for NaV1.7 binding, both doses of ASOs provided similar knockdown of *Scn9a* mRNA at lumbar DRGs and cord (Figure 4, E and F). Of note, changes in motor function for these 2 doses were observed, but only during a 24-hour period after ASO delivery and absent from day 1 to 14 after injections (Supplemental Figure 8, A and B).

As opposed to what was noticed at the DRG level, the NaV1.7 ASO treatment could not decrease the 488PTx-II binding in the sciatic nerves (Figure 5, A and C). Neither ASO dose tested interfered with binding of PGP 9.5 (control) (Figure 5, A and B) and 488PTx-II (Figure 5, A and C), as compared to mismatch-treated controls. In addition, no changes were detected in *Scn9a* mRNA (Figure 5D).

## Discussion

The relevance of NaV1.7 for nociceptive processing comes from human studies reporting pain sensitivity covarying with gain- or loss-of-function mutations associated with the *Scn9a* gene (4, 28–34). These findings have been recapitulated in preclinical studies where NaV1.7 functionality has been linked with inflammatory and neuropathic pain phenotypes (10, 27, 35–40). In the present studies, we undertook a systematic assessment of the dose-dependent effects of an intrathecal ASO targeting NaV1.7 on pain behaviors, mRNA reduction in DRGs and spinal cord, and on NaV1.7 binding. The discussion of our findings will be addressed in specific topics below.

*ASO specificity and effectiveness profile*. The specificity of our intrathecal NaV1.7 ASO targeting was established in ex vivo and in vivo studies where the active ASO but not its mismatch displayed prominent selectivity for specific knockdown of NaV1.7 mRNA and with no interference with other DRG NaV channels (NaV1.1, -1.2, -1.6, -1.8, and -1.9).

Consistent with the previous use of NaV1.7 loss-of-function mutations, neuraxial transfection, or ASO treatment (11, 14, 41–44), intrathecal NaV1.7-targeting ASO administration in rats dose-dependently reduced facilitated states of nociceptive processing as assessed by its effects on phase-2 formalin flinching, and mechanical thresholds assessed in carrageenan-treated animals. Similar doses of intrathecal ASO mismatch were without effect.

Importantly, the intrathecal ASO–mediated KO of *Scn9a* mRNA had little or no effect on acute nociception (e.g., phase 1 of the formalin) or low threshold mechanical-sensory perception (e.g., absence of effect on light touch, motor function, or proprioception). Of note, the expression of NaV1.7 in the ventral horn (motor efferents) is minimal/none in comparison with other NaV subunits (1.1 and 1.6), with most motor neurons lacking expression of NaV1.7 (9, 45).

On the other hand, previous work has reported transient effects on motor function with intrathecal ASO (46–48). The mechanism of this transient action is not certain. Previous work with other ASOs has suggested that such transient actions may reflect the transient chelation of calcium by the ASO that would eventually impact sensorial communication in the spinal cord, and further deficits in proprioception and motor function (47, 49). Overall, the consensus in the literature is that these effects have been observed in the absence of any specific ASO targeting (e.g., NaV1.7) and while they are often referred to as “neurotoxicity,” the effects were transient and, importantly to our observations, completely absent after 24 hours and during the subsequent assessments of the pain phenotype.

While the block of the acute algogenic effect of intraplantar OD-1 in the intrathecal ASO–treated rat is consistent with a KO of the target binding site, we did not see a loss of NaV1.7 binding in the peripheral nerve. An alternative explanation is that OD-1 activated the afferents but the loss of NaV1.7 in the soma of primary afferents or central terminals prevented the nociceptive afferent traffic to the second-order neuron (25). The duration of these intrathecal ASO effects is typically long-lasting and were not systematically examined by changes in threshold out to at least 4 weeks. Alternatively, as the KO mRNA is incomplete, residual synthetic capacity and preferential transport to the terminal may lead to residual channel expression in the terminals.

In our hands, male and female rats treated with the NaV1.7 ASO optimal dose (1000 μg/10 μL) both exhibited substantial increases in mechanical thresholds assessed through Randall-Selitto apparatus. These observations covary with our ASO uptake findings in the DRGs, where ASO particles were detected in large and small neurons. Both cells are known to express NaV1.7 (5) and contribute to mechanical pain associated with deep-pressure stimulus (10, 27). The analgesic effect upon mechanical compression–evoked withdrawal thresholds is consistent with the likelihood of a facilitated state arising from a degree of local injury resulting from repeated compression of paw soft tissue. This injury would be sufficient to induce a pain state through a modest local injury (e.g., plasma extravasation) that induces a state of facilitated processing as compared with the brief exposure resulting from an acute thermal stimulus.

The delivery in rats of the optimal NaV1.7 ASO doses having significant effects upon *Scn9a* mRNA and producing analgesia was well tolerated. Following ASO but not mismatch, while there was unexpectedly a transient dose-dependent reduction in rotarod performance, no effects were observed on the hind paw acute thermal response and there were no changes in body weight over the following 14 days (as shown in Supplemental Figure 8, A–E). In these rats, there were no significant changes in IBA-1 (microglia) or GFAP (satellite cells/astrocytes) mRNA in comparison with mismatch and PBS controls (as shown in Figure 1, E and F). Notably, previous reports have reported the moderate appearance of DRG and spinal pathology secondary to the catheter placement (50, 51).

*Neuraxial ASO distribution and uptake*. Neuraxial cerebrospinal fluid (CSF) represents a route for intrathecal solutes to redistribute. However, this space, while subject to local pressure gradients, presents as a poorly distributed volume with bulk rostro-caudal reflective of injection volumes (52). Assessment of the presence of ASO particles revealed that intrathecal delivery of either mismatch or NaV1.7-targeted ASO lead to rostral distribution of ASO particles after bolus delivery, showing a progressive gradient with lower levels at the thoracic and cervical levels relative to the lumbar cord and DRGs. In addition, uptake was evident in cells at brainstem and forebrain structures, including the cerebellum, cerebral cortex, hippocampus, thalamus, and olfactory bulbs. The intrathecal ASO particles in the intrathecal fluid displayed movement along the root sleeves, with cellular uptake into large and small DRG neurons (53). At the spinal level, the pia is present as an exclusion barrier for larger molecules and solutes (e.g., high molecular weight dextrans and adenoviruses) (54). As indicated here, given their presence in the spinal parenchyma, the pia does not present such a barrier for these low molecular weight ASOs (55, 56). In tissue, ASOs associate with specific cell surface membrane proteins that mediate internalization, and such productive internalization is observed in both glial and neuronal cell types (57, 58). These observations highlight our findings, as ASO particles were detected in large- and small-sized neuronal populations in DRGs.

*Localization of intrathecal ASO effects on NaV1.7 expression*. The rostro-caudal gradient of ASO distribution resulting from the lumbar bolus delivery covaried with the observed reduction in the *Scn9a* mRNA observed in DRGs for any given dose. Thus, as seen in the dose-response analysis, the greatest silencing occurred at the lumbar level of injection, with progressive reduction in the target DRG effects at rostral levels. These observations are supported by the shift to the right in the dose-response curve, showing a progressive increase in the IC_50_ across the spinal levels.

Importantly, increasing the intrathecal ASO dose (with a fixed volume) resulted in a greater rostro-caudal distribution of NaV1.7 mRNA reduction. These results recapitulate distributional transfection studies after intrathecal AAV injection in the rat model with a lumbar catheter and in the mouse after a percutaneous lumbar bolus, where a robust lumbar to cervical gradient of DRG transfection was observed (11, 59). Of note, in these models with a fixed injection volume the rostro-caudal degree of transfection increased as a function of concentration (e.g., viral titer).

An important issue that is not often considered is that *Scn9a* mRNA is observed in the dorsal horn (6). While it has been suggested that functional NaV1.7 channels are present in substantia gelatinosa neurons, the source of these channels is speculative (60). A study by Alles et al. suggested that there is transport from the afferent terminal to the second-order neurons (6), while others have reported low levels of NaV1.7 mRNA in the spinal dorsal horn (45, 61). In our work, we note that the dorsal horn mRNA was effectively abolished by even low levels of intrathecal NaV1.7 ASO, which suggests potential effects on non-afferent neuraxial components occurring as a result of movement of the ASO though the pial barrier. Recent work by Shiers et al. (60) suggested that NaV1.7 may be expressed in neurons that project along ascending pathways. However, as the authors stated, no convincing evidence of NaV1.7 expression in the soma of laminae I–V neurons was found. Further detailed investigations will be of great interest to unravel such expression in these links known to be relevant to the trafficking of nociceptive information.

*ASO dose effects on pain behavior*. In the present studies, we show that the intrathecal injection of ASOs at doses between 100 and 3000 μg led to a dose-dependent silencing of *Scn9a* mRNA in lumbar (L1–L5) DRGs harvested 14 or 28 days after the single bolus ASO delivery at the spinal level in rats. In all behavioral models, the lowest intrathecal ASO dose that resulted in a significant reduction in the measured nociceptive behavior as assessed by high-intensity mechanical compression, phase 2 formalin flinching, and inflammation induced tactile allodynia, was 1000 μg. Assessment of the relationship between *Scn9a* mRNA reduction and intrathecal ASO dose similarly revealed a close covariance, with the maximum reduction in the lumbar DRGs being in the range of 50%–75%.

We note that for doses such as 100 and 300 μg, the degree of mRNA suppression was often similar, but the analgesic effects were limited to the higher dose concentrations. This observation brings to the forefront the appreciation that increasing the injected dose by increasing concentration (vs. increasing injection volume) not only increases the local tissue exposure at the injection site but also results in delivering a higher solute concentration to targets that lie more distal to the injection level, which may contribute to the nociceptive signal underlying the pain state.

It is important to note that for models such as formalin, evidence of terminal activation as measured by, for example, c-Fos leads to greatest activation in the L3–L5 segments but significant expression may be observed at segments ranging from S1 to T13 (62–64). Furthermore, in acute models the primary activation is superficial (e.g., the substantia gelatinosa), whereas in more chronic exposures the activation shifts to the deep dorsal horn (62–64).

Accordingly, the concentration at the local DRG site may be maximum with a given dose (concentration) but when volume of delivery remains fixed, the higher concentrations may lead to a broader rostro-caudal distribution of NaV1.7 KO and further affect nociceptive signaling. This organization may reflect the role played by NaV1.7-expressing circuits in the spinal parenchyma. Thus, the differences in pain behavior seen between the ASO doses could likely reflect the role of these NaV1.7-expressing circuits in modulating the ascending pain pathway in the formalin test, where ASO higher concentrations such as 1000 μg would effectively mitigate the activity of such nociceptive ascending circuits.

*Effects of the pain state on ASO knockdown*. We observed a more effective knockdown of *Scn9a* mRNA by the ASO optimal dose (1000 μg/10 μL) in DRGs at inflammatory pain triggered by carrageenan insult rather than formalin. These different profiles of knockdown varying according to stimulus may reflect different degrees to which NaV1.7 signaling is regulated. This apparent increase in knockdown may reflect several linked events. NaV1.7 channel transcription can be enhanced by cellular activity and by the local effects of inflammatory mediators released in the DRGs from macrophages and satellite cells (e.g., TNF-α or GM-CSF) (65). These products can lead to increased NaV1.7 transcription and further expression and in this case, promote the effects of ASO on mRNA. This is not surprising, as ASOs bind to nascent RNA during transcription (66). Accordingly, ASO effects can be increased, by the rate at which genes are being transcribed (67). On that note, NaV1.7 undergoes activity-dependent internalization and turnover (68) and transport to the membrane (e.g., CRMP2), which may be altered in the face of afferent traffic due to posttranslational changes (69, 70). So, these functional changes would make ASOs appear more efficacious in the face of injuries leading to afferent neuron activation. Lastly, it is from our understanding that formalin represents a relatively brief interval (<60 minutes) of high-frequency activation (71). In contrast, carrageenan represents an ongoing inflammation-mediated sensitized state in which persistent afferent traffic is initiated over intervals of 12–24 hours (72, 73). We suggest that the difference in time course of these 2 stimulus conditions contributes to the explanation for these differences in ASO activity.

Although we performed the analysis from DRG ASO uptake only 14 days after intrathecal delivery, the differences in knockdown profile seen in DRGs harvested at different endpoints also reflect a kinetic relationship between the bioavailability of oligonucleotides in the cell compartment and regulation of target mRNA. Assuming a drug concentration paradigm, such differences seen for ASO knockdown between day 14 and day 28 endpoints suggest that the ASO would better regulate genomic alterations involving *Scn9a* at 14 days rather than 28 days, and thus effectively mitigate pain behavior depending on NaV1.7 early on.

*Effects of NaV1.7 ASO on other sensory systems*. It has been reported that high doses of systemic NaV1.7 inhibition result in dose-dependent decreases in blood pressure and heart rate, suggesting an effect of NaV1.7 blockers on the sympathetic system (74). We noticed a minor but significant decrease in *Scn9a* mRNA in SCG after intrathecal delivery of ASO optimal dose. As previously discussed, these results reinforce the ability of ASO solutes, at a determined concentration, to be distributed through the CSF axis in a rostral manner and later be taken up at the SCG. The mechanisms by which ASOs reach the SCG are unknown. One possibility is that ASOs could reach the SCG via diffusion through segmental vessel systems that do contribute to CSF clearance and supply the SCG as well (75).

On the other hand, phosphorothioate ASOs, such as the one used in this work, can undergo cellular uptake by associating with membrane proteins such as clathrins (57), and further merge into endocytic pathways to regulate downstream RNA translation (57, 76, 77). SCG neurons do exhibit clathrin-mediated endocytosis (78). Thus, a consequence of such ASO rostral distribution could be a direct ASO uptake through the cell bodies of preganglionic neurons that relay on the lateral horn of the spinal cord (79), and a further decrease in excitability mediated by NaV1.7 knockdown. The effects of this would be noticed in autonomic reflexes that are influenced by spinal cord activity such as blood pressure (80) and coughing reflex (81). In our studies, we have not assessed such changes in the ASO-treated rats, as our focus here has been the role of intrathecal ASOs in neuraxial DRG NaV1.7 expression and the relationship between neuraxial NaV1.7 KO and nociceptive processing. However, in this regard, we would note that behaviorally relevant signs of hypotension (e.g., behavioral suppression and signs of behavioral distress) were not observed. The only time we observed any adverse behavioral effects were on motor function during the initial 24 hours after ASO delivery and when NaV1.7 KO had not been incurred. Over the 4-week post-ASO period, normal weight gain, behavior, and motor function (gait, activity) were routinely observed.

We would parenthetically note that while intrathecal small molecules and ASOs reach the systemic circulation, the impact of this redistribution on peripheral tissue is dependent on the total intrathecal dose delivered and the initial concentrations produced by the redistribution into the much larger peripheral blood volume and, its rapid clearance by the liver and metabolism (51, 82, 83). Using the widely expressed *Malat1* mRNA in spinal cord and liver, it has been shown that the intrathecal dose leading to almost complete KO of *Malat1* mRNA in spinal cord led to a less than 17% reduction in *Malat1* mRNA in the liver (83).

*Quantitative assessment of NaV1.7 antagonist binding*. 488PTx-II is a NaV1.7 antagonist that carries the ATTO488 azide tag. This allows fluorescence microscopy imaging at 488 nm (20). This toxin is highly selective for NaV1.7 binding at the external voltage sensing site, displaying minimal affinity for other NaV channels (20). To further address this issue, we examined (as shown in Supplemental Figures 6 and 7) the covariance of signals in HEK cells transfected with NaV1.7-HaloTag channels and 488PTx-II. In ex vivo studies, consistent with preferential binding to nociceptors, the 488PTx-II binding in DRG cultures showed reliable binding in small (<200 μm^2^) but not larger cells that are not typical nociceptors in the rodent (84). In our binding studies with the rat DRG cell culture system, we observed that knockdown of *Scn9a* mRNA, using the optimal intrathecal ASO dose, resulted in a significant reduction in 488PTx-II binding in comparison with mismatch controls, both as measured by fluorescence intensity (reflecting binding in cells still expressing the channels) and the number of cells expressing NaV1.7 binding, suggesting that some cells experience a full KO of the channel. These data confirm the utility of the 488PTx-II binding in defining the presence of membrane expression NaV1.7 channels, while the ASO studies show the potential functional outcome of a determined dose inside the DRG sensory system. On the other hand, it is important to note that NaV1.7, as with many proteins, is synthesized in the cell body packaged into vesicles and undergoes transport to the membrane, afferent axon, and the terminal (5, 85). We note that even in the DRGs at the highest ASO doses we do not see 100% suppression of either mRNA or 488PTx-II binding. We believe it is likely that the residual NaV1.7 binding reflects the ongoing movement of NaV1.7 protein to the afferent axon and terminal.

*Covariance of analgesia with ASO doses and NaV1.7 binding*. We sought to systematically assess the ASO dose and NaV1.7 binding covariance using the intrathecal delivery of the NaV1.7 ASO in the hind paw formalin flinching model. Here we observed that the intrathecal 1000 μg group displayed a highly significant suppression of phase 2 flinch as compared with the mismatch or the 300 μg NaV1.7 ASO. In parallel, the ipsilateral NaV1.7 binding on DRGs of the 1000 μg, but not the 300 μg rats, was significantly reduced as compared with mismatch controls. Strikingly, in both these groups (300 μg and 1000 μg), the ipsilateral reduction in NaV1.7 mRNA was significantly reduced in comparison with the mismatch. This difference between mRNA and channel expression suggests that the binding expression presents as a closer covariance to the actual analgesic variable than the mRNA expression itself. One explanation may reflect the time required for turnover of the membrane-expressed channels (as we believe to be measured by the 488PTx-II binding). As noted, these studies examined binding and mRNA at 14 days and studies on NaV1.7 turnover suggest that this event may occur roughly in a time frame of 5 days (86). How the ASO dose would impact NaV membrane turnover is not clear. It is notable that the NaV1.7 channel displays turnover, and its replacement involves the role of a transporter protein (CRMP2) (87), although how these ASOs affect the activity of such a transporter is not known. It is important to note that ASO uptake in the spinal cord follows a gradient pace, with lumbar absorption preceding thoracic and cervical levels (56). In this regard, as discussed above, a second possibility may be that the lower ASO dose resulted in a more limited rostro-caudal distribution of target engagement and the lack of effect at the lower ASO dose reflects a lack of KO at distal DRGs or parenchymal tissue that is contributing to the formalin flinching.

*Peripheral nerve NaVs and ASO effects*. A point of interest was the observation that NaV1.7 binding was not lost in peripheral nerves 14 days after intrathecal ASO administration. This presence of NaV1.7 binding in ASO-treated nerves could indicate a potential limitation of ASOs. In this regard, NaV1.7 ASOs may exert their effects by blocking spinopetal transmission at the DRG level, thereby reducing the afferent signal reaching spinal levels. Consequently, as demonstrated in the present studies, NaV1.7 ASOs exhibit an analgesic profile predominantly in facilitated pain states. These phenotypes have been suggested to show increased soma NaV1.7 expression (88).

### Concluding remarks

The present studies demonstrate the efficacy of intrathecal ASO selectively targeting neuraxial NaV1.7 in producing a robust dose-dependent analgesia as measured in models of facilitated processing. Our studies assessing membrane expression of the channel using a fluorophore-labeled ligand binding at an extracellular site on the NaV1.7 protein revealed the importance of assessing the expression of the membrane-expressed channel versus mRNA. The presence of mRNA in the spinal parenchyma raises the possibility that a component of the efficacy of the NaV1.7 reduction reflects these effects on preventing the role of NaV1.7 in the dorsal horn systems. Studies carrying further investigations related to the contribution of such spinal dorsal horn systems in NaV1.7 pharmacology will be of great importance for understanding the relevance of NaV1.7-dependent spinal circuits for the development of facilitated pain states.

## Methods

Experimental details are described in the Supplemental Methods.

### Sex as a biological variable.

The study included both male and female rats. Most experiments were conducted in males. It is our understanding that further research is needed to assess the effects of ASOs on NaV1.7 channel traffic in females, as sexual dimorphism has influenced outcomes in some pain models that rely on NaV 1.7 activation (89).

### Statistics.

The analysis of the results was performed using GraphPad Prism v10 software. The normal distribution of values in each analysis was assessed with the D’Agostino Pearson, Shapiro-Wilk, or Kolmorogov-Smirnov test, according to the sample size. When a non-parametric statistical test was performed, the Mann-Whitney test was used to compare 2 means. When the comparison involved more than 2 means, the Kruskal-Wallis test was performed with Dunn’s post hoc test to determine whether there were differences between the groups analyzed. If the values followed a normal distribution, parametric statistical tests were done. The 2-tailed Student’s *t* test was selected when 2 means were compared. When the comparison involved more than 2 means, a 1-way or 2-way analysis of variance (ANOVA) was performed according to the experimental design. When the level of significance indicated a statistical difference between the analyzed means, Tukey’s or Dunnett’s test was used to compare the different analyzed groups. The level of significance adopted in each analysis of the results was a *P* value of less than 0.05.

### Study approval.

All experiments were carried out according to protocols (protocol number S00137R) approved by the Institutional Animal Care Committee (IACUC) of UCSD.

### Data availability.

The data presented in this manuscript are available with the corresponding author upon reasonable request. Values for all data points in graphs are reported in the Supporting Data Values file.

## Author contributions

KM: conceptualization, methodology, investigation, data curation, data analysis, writing original draft. JBPL: methodology, investigation, writing original draft. BN: methodology, investigation, data curation, data analysis, writing original draft. YA: methodology and investigation. CEMU: methodology, data curation, data analysis, writing original draft. SJ: methodology and data curation. SY: investigation and data curation. JM: investigation and data curation. VA: formal analysis, writing review and editing. SAD: formal analysis and writing-review and editing. CK: methodology and data curation. MD: investigation and data curation. PA: conceptualization, methodology, investigation, data curation, and data analysis. BF: conceptualization, methodology, investigation, data curation, data analysis, and writing-review and editing. CM: conceptualization, methodology, investigation, data curation, and data analysis. HBK: conceptualization, methodology, investigation, data curation, and data analysis. KD: conceptualization, supervision, writing-original draft, writing-review and editing. HTZ: supervision, project administration, resources, writing-review and editing. TY: conceptualization, supervision, project administration, resources, writing-original draft, writing review and editing.

## Conflict of interest

BN, BF, HBK, and HTZ are employees of IONIS. PA and CM are former employees of IONIS.

## Funding support

This work was partially funded by IONIS Pharmaceuticals.

## Supplementary Material

Supplemental data

Supporting data values

## Figures and Tables

**Figure 1:**
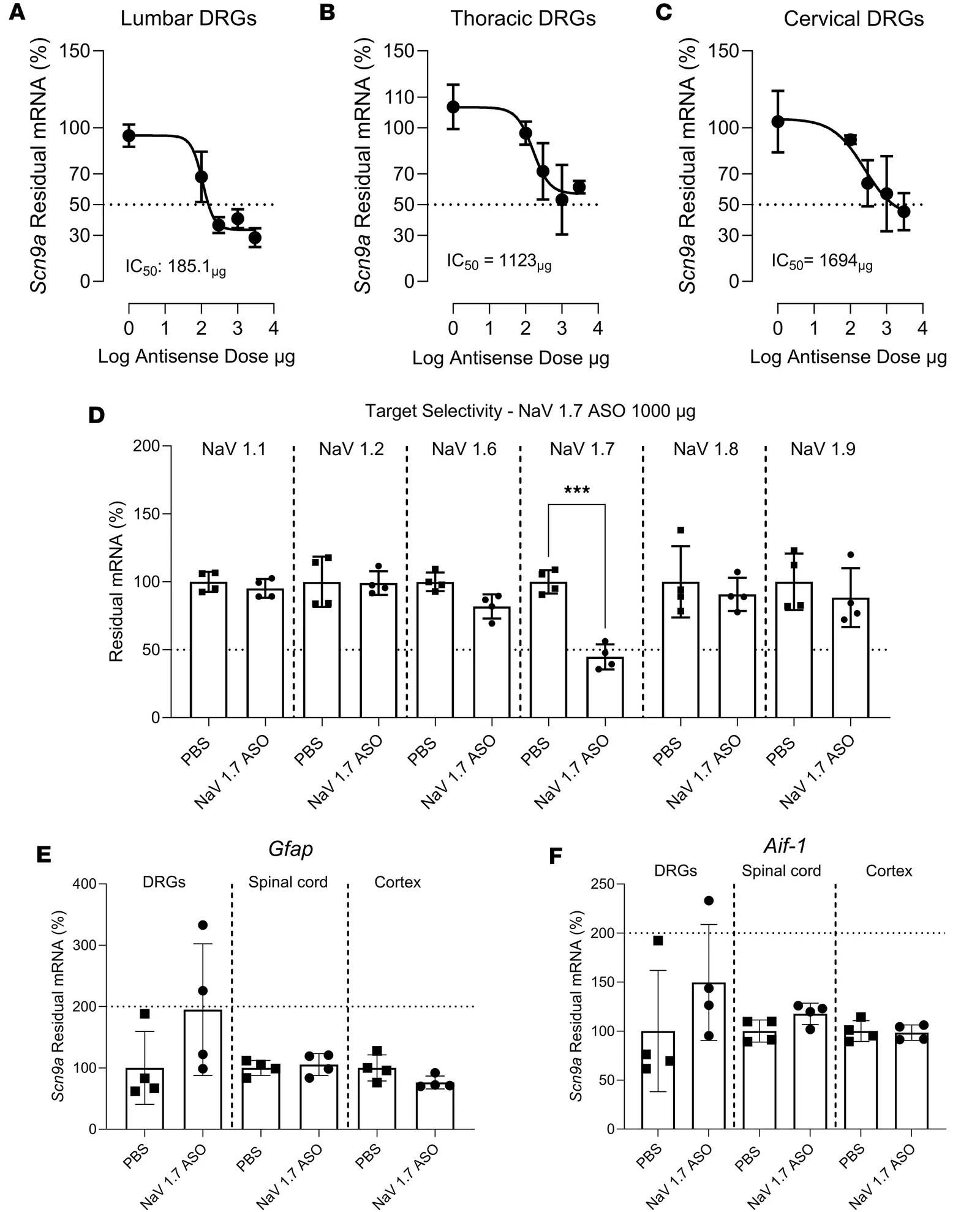
Dose-dependent knockdown of *Scn9a* mRNA in dorsal root ganglia across the spinal cord axis. (**A**–**C**) Dose-response analysis from the intrathecal injection of NaV1.7 ASO (100–3000 μg in 10 μL) showing dose-dependent knockdown of *Scn9a* in lumbar, thoracic, and cervical DRGs (In **A**: sum square [SS] = 2125; degrees of freedom [DF] = 16; *F* = 6.275. In **B**: SS = 4624; DF = 17; *F* = 4.640. In **C**: SS = 6153; DF = 18; *F* = 6.484). (**D**) The effective dose of NaV1.7 ASO (1000 μg/10 μL) selectively targets NaV1.7 and promotes knockdown of *Scn9a* mRNA at the lumbar DRGs without interference in other voltage-gated sodium channels subtypes. (**E** and **F**) Assessment of DRG, spinal cord, and brain cortex toxicity related to the optimal dose of NaV1.7 ASO (1000 μg/10 μL). This dose did not lead to significant increase in glial activity markers *Aif-1* (**E**, IBA-1 protein; macrophages/microglia) and *Gfap* (**F**, GFAP protein; satellite glial cells/astrocytes) in DRG, spinal cord, and cortex samples of ASO animals in comparison with PBS controls. Results shown as mean ± SD. ***P < 0.001 by Student’s *t* test (**D**–**F**).

**Figure 2:**
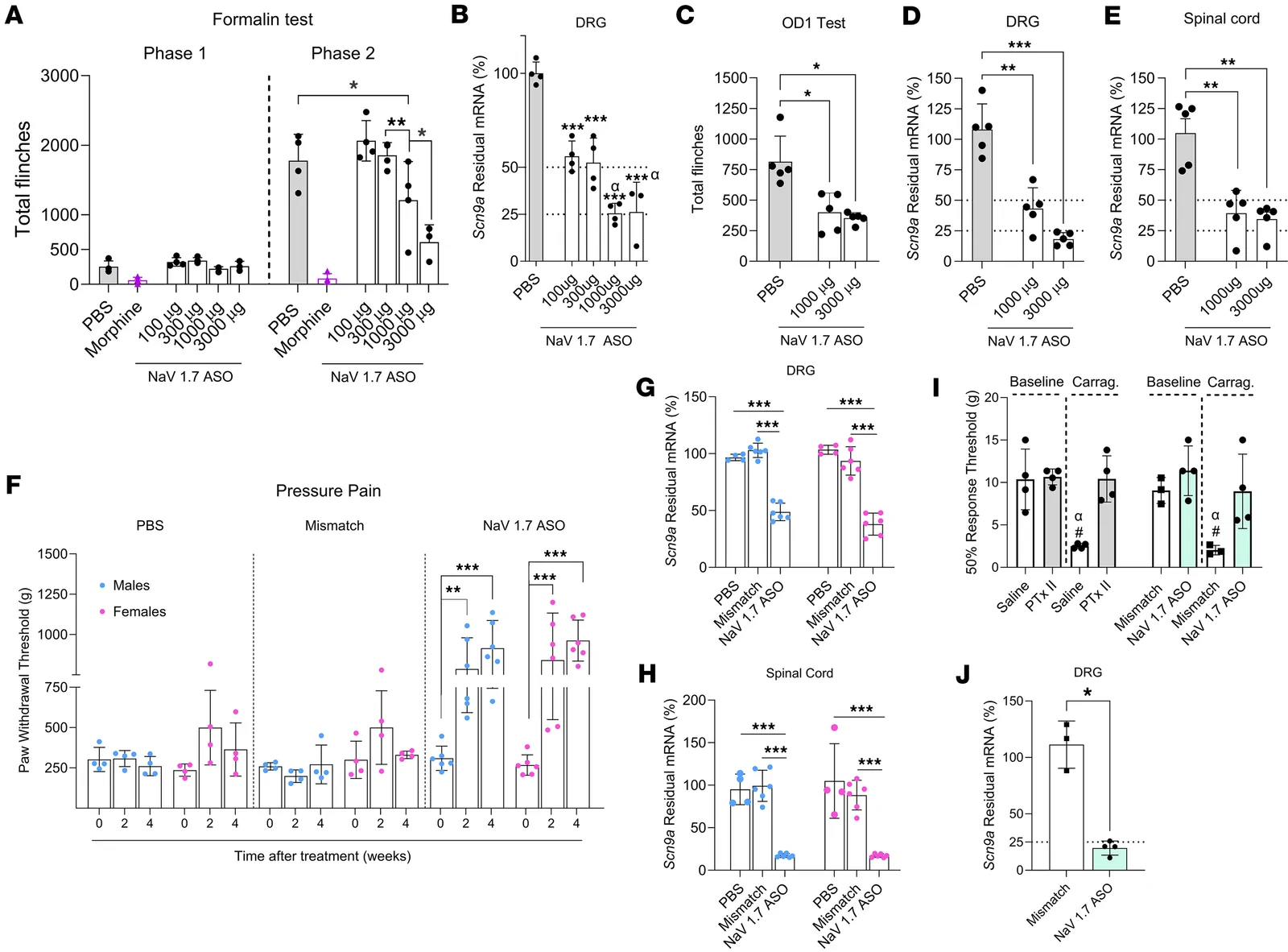
ASOs targeting NaV1.7 affect nociceptive behaviors. (**A** and **B**) The intrathecal injections of different NaV1.7 ASO doses (100–3000 μg in 10 μL) significantly knocked down *Scn9a* mRNA in DRGs and decreased formalin second phase. In **B**: ^α^*P* < 0.05, compared to NaV 1.7 ASO 300 μg group. (**C**) The nociceptive behavior evoked by the selective NaV1.7 agonist, OD-1, was significantly affected by the intrathecal knockdown of NaV1.7 with 1000 and 3000 μg ASO doses. (**D** and **E**) These doses tested had similar outcomes in decreasing the flinching behavior triggered by intradermal injection of OD-1, with similar degrees in mRNA knockdown at the DRG (**D**) and spinal cord (**E**). (**F**) On a 4-week time course, the intrathecal NaV1.7 ASO markedly increased the paw withdrawal thresholds in male and female rats at weeks 2 and 4 after the ASO delivery. (**G** and **H**) These events are correlated with a knockdown of *Scn9a* at both DRGs (**G**) and spinal cord (**H**) in comparison with mismatch and PBS controls. (**I**) In an extra set of experiments, the intrathecal NaV1.7 ASO (1000 μg/10 μL, intrathecal, 14 days prior to testing) replicates the analgesic profile of the NaV1.7 antagonist 488PTx-II (2 μg/10 μL, intrathecal) in the carrageenan model (100 μg/50 μL, subcutaneously, right hind paw). ^α^*P* < 0.05, compared to PTx-II or NaV 1.7 ASO groups during carrageenan testing. (**J**) Again, the effects of NaV1.7 ASO correlates with a significant knockdown in *Scn9a* mRNA in the ipsilateral L3–L5 DRGs harvested from mismatch and NaV1.7 ASO immediately after testing. Results shown as mean ± SD. *P < 0.05; **P < 0.01; ***P < 0.001 for comparison with PBS by 2-way ANOVA with Tukey’s post hoc test (**A**) or 1-way ANOVA with Tukey’s post hoc test (**B**–**E**).

**Figure 3:**
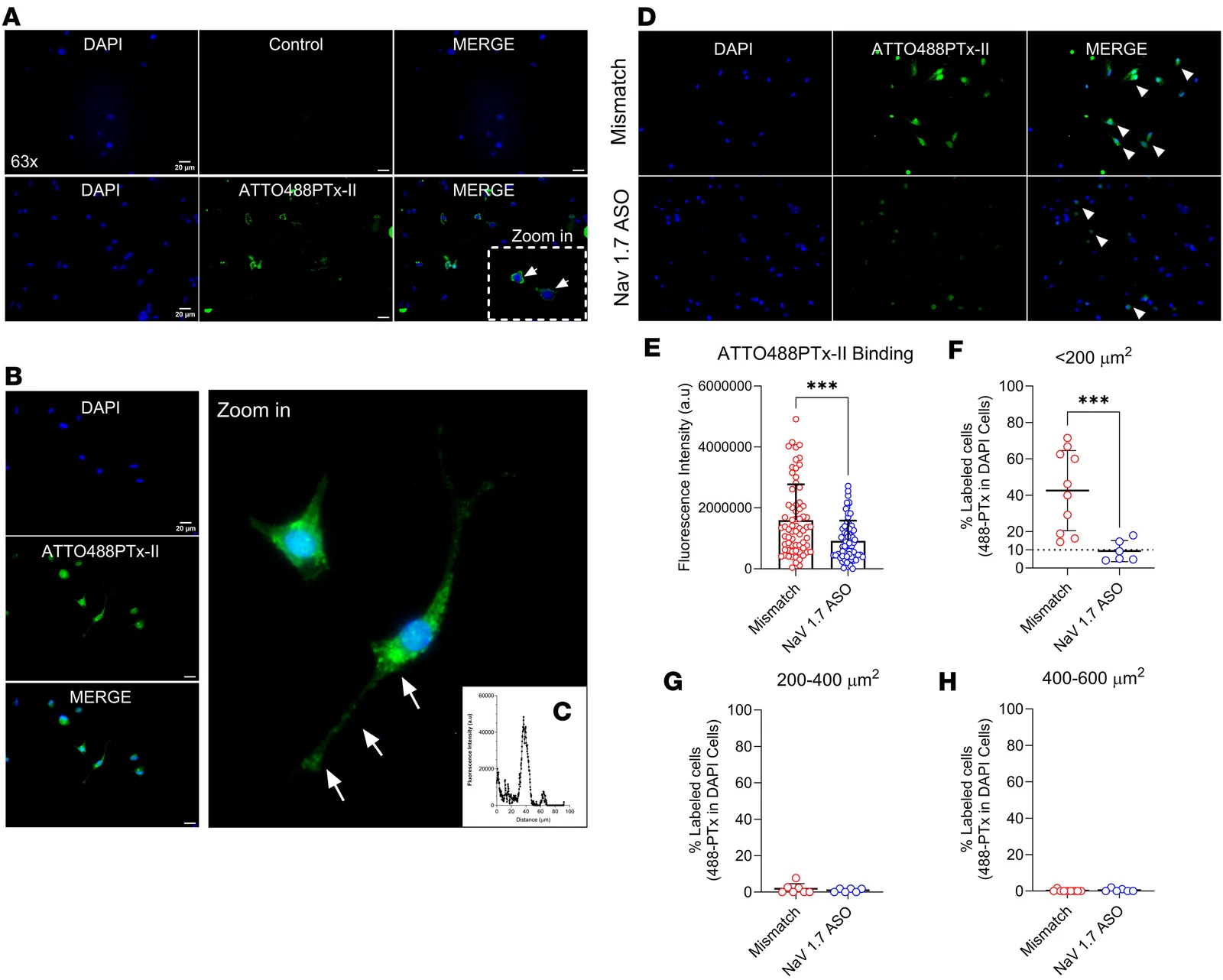
NaV1.7 ASO reduces the binding of 488PTx-II predominantly in small DRG neurons. (**A**) Naive DRG neurons labeled with 488PTx-II display visible fluorescence signal in comparison with non-treated controls. (**B** and **C**) These naive DRG cell cultures labeled with 488PTx-II (**B**) display punctate signal towards the neuronal soma and axons (**B**, zoom-in section white arrows, and **C** for densitometry assessment). Scale bars: 20 μm. (**D** and **E**) The intrathecal injection of the NaV1.7 ASO optimal dose (1000 μg/10 μL) led to a significant decrease in 488PTx-II binding in DRG neurons. (**D**) Scale bars: 20 μm. In **E**, mismatch = 73 cells; Nav1.7 ASO = 72 cells. (**F**–**H**) In small neurons, the fraction of cells expressing NaV1.7 channels is significantly reduced in the presence of ASO. Mismatch group: *n* = 11 fields analyzed; DAPI cells = 236; 488PTx-II cells = 65. NaV1.7 ASO group: *n* = 6 fields analyzed; DAPI cells = 786; 488PTx-II cells = 66. Results shown as mean ± SD. ***P < 0.001 by Mann-Whitney test (**E**) or Student’s *t* test (**F**).

**Figure 4:**
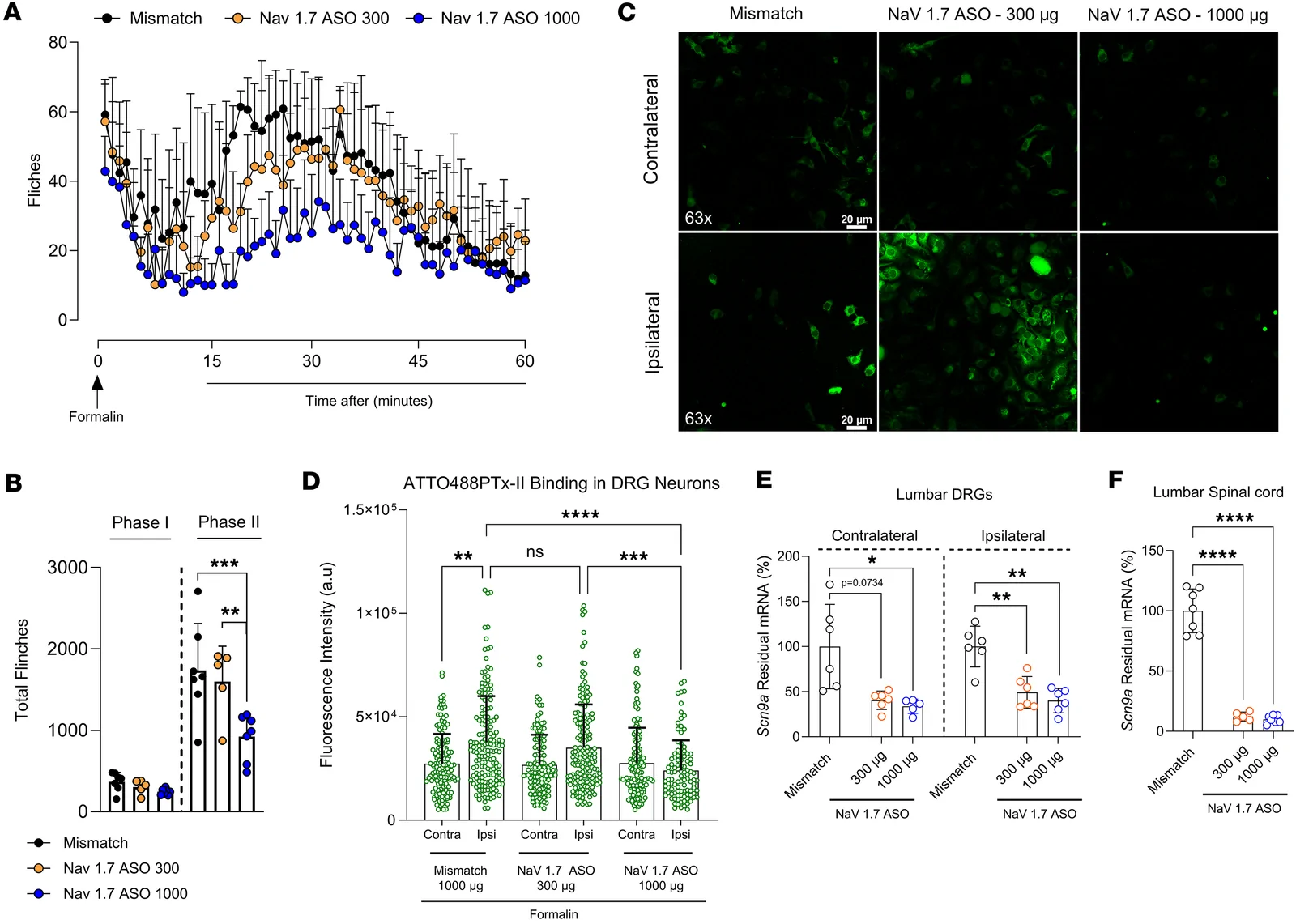
NaV1.7 ASO affects formalin behavior in a dose-dependent way with a different profile for 488PTx-II binding. The lumbar intrathecal delivery of NaV1.7 ASO (300 or 1000 μg in 10 μL) using spinal catheters led to a different response in formalin nociceptive behavior. (**A**, **C**, and **D**) The injection of formalin (2.5%, 50 μL, intradermal) at the hind paw led to flinching responses (**A**), with an increase in 488PTx-II binding in ipsilateral DRGs from mismatch animals (**C** and **D**). A similar profile was followed by the NaV1.7 ASO smaller dose (**A**–**D**). The optimal dose of NaV1.7 ASO (1000 μg/10 μL) was able to reduce the formalin-induced flinching at phase 2 (**A** and **B**) and decrease 488PTx-II binding in ipsilateral DRGs in comparison with mismatch and the smaller antisense dose (**C** and **D**). At lumbar DRGs (**E**, contralateral and ipsilateral) and at the lumbar spinal cord (**F**) a significant reduction in *Scn9a* mRNA is seen for both NaV1.7 ASO doses in comparison with mismatch controls. Scale bars: 20 μm. Results shown as mean ± SD. In **D**, mismatch (Contra = 147 cells/Ipsi = 161 cells), NaV1.7 ASO 300 μg (Contra = 125 cells/Ipsi =166 cells), Nav 1.7 ASO 1000 μg (Contra = 147 cells/Ipsi = 101 cells). *P < 0.05; **P < 0.01; ***P < 0.001; ****P < 0.0001 by 2-way ANOVA with Tukey’s post hoc test (**B**), Kruskal-Wallis with Dunn’s post hoc test (**D**), or 1-way ANOVA with Dunnett’s post hoc test (**E** and **F**).

**Figure 5:**
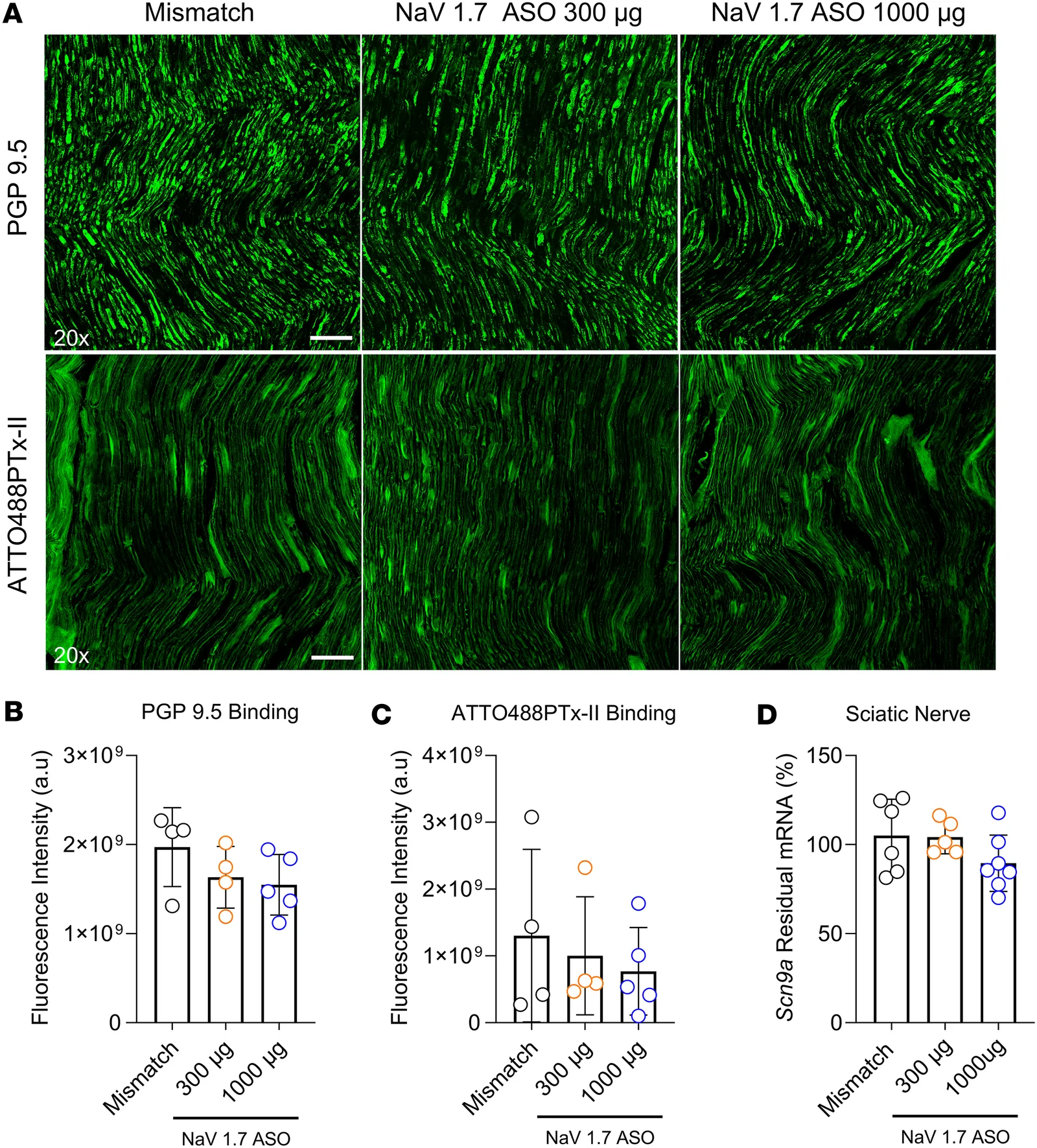
NaV1.7 ASO does not decrease 488PTx-II binding in sciatic nerves. (**A** and **C**) The lumbar intrathecal delivery of NaV1.7 ASO (300 or 1000 μg in 10 μL) using spinal catheters did not lead to a decrease in binding of 488PTx-II in sciatic nerves harvested from rats 24 hours after being ipsilaterally stimulated with formalin (2.5% in 50 μL, intradermal) in the hind paw. Original magnification (**A**), ×20. (**B**) Quantification of PGP9.5 binding in sciatic nerves of mismatch, and NaV 1.7 ASO groups. (**D**) *Scn9a* mRNA at the sciatic nerve was not affected by the ASO treatment at both doses tested. Results shown as mean ± SD. Significance was assessed using the Kruskal-Wallis test with Dunn’s post hoc test (**B** and **C**) or 1-way ANOVA with Dunnett’s post hoc test (**D**).

## Version 1 10/22/2026

Electronic publication
