# Supplementary material for: Characterizing NaV1.7 antisense analgesia through antagonist binding, mRNA expression, and neuraxial distribution
Source: JCI Insight. 2026 Oct 22;11(20):e196154. doi: 10.1172/jci.insight.196154 (PMC13726551; doi:10.1172/jci.insight.196154)
Supplement: Supplementary file 1 [file jciinsight-11-196154-s180.pdf]

1. METHODS

1.1. Animals and intrathecal drug delivery

Male Sprague–Dawley rats (200–250 g) were prepared with lumbar intrathecal catheters as previously described by (83) and (84). For dosing and distribution studies the Mazur et al. (84) technique was used to deliver ASO to the lumbar cord to study the ability of the NaV 1.7 antisense in knock-down *Scn9a* mRNA in dorsal root ganglia (DRGs) across the spinal column segments. The same surgical technique was used to perform the pharmacological validation and selectivity assessment from the antisense optimal dose.

After defining the optimal dose for the antisense, lumbar delivery of ASO was performed using the Yaksh technique (85) to assess the effects of *Scn9a* mRNA knock-down and reduced NaV 1.7 binding on the formalin induced flinching.

The therapeutic protocol for the delivery of ASOs started 7 days after surgery. Two antisense oligonucleotides (IONIS Pharmaceuticals Inc., Carlsbad, California, USA) were used. NaV 1.7 ASO is an ASO that targets the *Scn9a* mRNA (NaV 1.7), where Mismatch is an ASO control (Detailed ASO characteristics is available at supplemental table-1). ASOs were dissolved in PBS without Calcium and Magnesium (Gibco™; 14190) and administered intrathecally as a single bolus injection (10μL) followed by a 10 or 30μL flush depending on the technique performed.

1.2. Assessment of nociception

In this study, three sets of nociceptive behavior were measured in rats. Initially, to evaluate the mechanical nociception two different methodologies were performed: the up-down method (86) or paw-pressure test (87). Thermal nociception was measured using a Hargreaves type device (88). Finally, the effects of IT treatments were conducted to evaluate the biphasic flinching nociceptive behavior triggered by unilateral hind paw intraplantar formalin (2.5%/50µL) or OD-1 (NaV 1.7 selective agonist; 500 nM; 50 µL). To measure flinching a lightweight metal band was placed around the hind paw receiving the formalin. Movement of the band in a detection field is collected automatically and presented as flinches/min (89). In all experiments, animals were set to acclimate for at least 60 minutes prior testing.

1.3. Defining the optimal dose of ASO

1.3.1. Dose-response analysis

To determine the ASO optimal dose, doses ranging from 100 to 3000µg/10µL were given intrathecally at 14 days prior tissue harvest. Mismatch was given at 1000µg/10µL. The dose paradigm and time frame for assessment of mRNA knock-down was based on previous studies published by our research group (84, 90, 91). After the timing required for ASO knockdown, the lumbar spinal cord, along with the lumbar, thoracic, and cervical DRGs, was harvested. In the same act, the trigeminal ganglia, superior cervical ganglia, and nodose ganglia were collected. The tissues were processed for *Scn9a* mRNA quantitative assessment through qPCR.

For the construction of the dose-response curves, the mismatch group was assumed as null dose in each analysis. The *Scn9a* mRNA in each group was then normalized to *Ppia*, a ubiquitously expressed housekeeping gene, and this was further normalized to the level measured in control animals that were administered PBS. The *Scn9a* residual mRNA data for each dose was obtained fixing PBS as ubiquitous control (100%) and relative value for each dose calculated as percentage of control. The values obtained were the *Scn9a* residual mRNA percentage for each given dose and used for non-linear regression analysis that were calculated with Graphpad prism software.

1.3.2. Selectivity assessment from antisense optimal dose

To demonstrate ASO selectivity, animals were previously treated with NaV 1.7 ASO, Mismatch or phosphate buffered saline (PBS) controls. On day 14 after injections, the animals were harvested for L3-L5 DRGs and mRNA quantitative assessment of several NaV subunits were performed, including *Scn1a* (NaV1.1), *Scn2a* (NaV 1.2), *Scn8a* (NaV 1.6), *Scn9a* (NaV 1.7), *Scn10a* (NaV 1.8) and *Scn11a* (NaV 1.9). All these channels are expressed in DRG sensory neurons and contribute to pain signaling (92–94).

1.3.3. Tolerability assays

Tolerability was assessed for the optimal dose of NaV 1.7 ASO by: I) immunohistochemistry (IHC) and II) quantitative measuring of mRNA for markers of glial activation: *Aif-1* (Ionized Calcium Binding Adaptor Molecule 1 -IBA-1) and *Gfap* (Glial fibrillary acidic protein-GFAP). The mRNA levels and/or IHC outcomes from these markers were measured in thoracic DRGs and spinal cords, and at the cerebral cortex

harvested on day 14 from NaV 1.7 ASO, Mismatch, and PBS injected animals.

1.3.3.1. Histopathology

We used immunohistochemistry to perform histopathology and analyze glial reactivity in DRGs, spinal cords, and Brain through IBA-1 staining. Gliosis was determined by following the score system in supplemental table 3, where activation was taken as an indicator of treatment related toxicity (95). Histopathological scoring as well as ASO uptake was analyzed by an investigator without knowledge of treatment.

Initially, slides were treated with Heat Induced Antigen Retrieval (HIER) with Ventana CC1 solution (Ventana, 950-500) for 64 minutes. The primary antibody was diluted with Discovery Antibody Diluent (Ventana, 760-108) and incubated for 1 hour at 37°C. The antibody was detected with UltraMap anti Rabbit Polymer detection (Ventana, 760-4315). The rabbit polymer was labeled with Ventana ChromoMap DAB kit (Ventana, 760-159). Images were scanned on a Hamamatsu S360 scanner at 20X resolution.

1.3.3.2. Assessment of motor function

For the assessment of motor function using the optimal dose of ASO, the animals were trained on the Rotarod for up to 5 days. During the training phase, animals were given a maximum of 4 trials daily for up to 5 days, or until successful completion of two 1-minute trials at 8 revolutions per minute (RPM). Each trial was separated by a minimum of 3 minutes to allow the animal adequate recovery. Baseline readings were acquired before any surgical procedure. On the test days, animals were placed on the rotating rod at 8 RPM, then the rod was accelerated from 8 to 40 RPM over 180 seconds. Each test-point consisted of two trials, separated by a minimum of 3 minutes. The duration

of time until the animal fell from the moving Rotarod was measured and recorded. For each time of assessment, the longer of the two trials was selected and included in the data set.

1.3.4. Antisense uptake studies

We analyze the ASO uptake in DRGs, Spinal cords, and Brain. Here, the tissue was fixed and then processed on a mouse brain protocol using a Sakura Tissue Tek tissue processor. After embedding, slides were cut at 4 μm, air dried overnight then dried at 60°C for 1 hour. Slides were stained with Rabbit polyclonal ASO (IONIS) antibody on a Ventana Ultra staining system. ASO slides were treated enzymatically with Trypsin (Sigma, T8003). The slides were then blocked with Endogenous Biotin Blocking Kit (Ventana, 760-050) and Normal Goat Serum (Jackson Immuno Labs, 005-000-121). The primary antibody was diluted with Discovery Antibody Diluent (Ventana, 760-108) and incubated for 1 hour at 37°C. The antibodies were detected with Biotin labeled Goat Anti-Rabbit secondary antibody (Jackson Immuno Labs, 111-005-003). The secondary antibody was labeled with DABMap Kit (Ventana, 760-124). Images were scanned on a Hamamatsu S360 scanner at 20X resolution.

1.3.5. Assessment of dose effectiveness on pain behaviors

The effectiveness of ASO optimal dose was analyzed along with several other ASOs doses in different models of nociception. The dosing and time frame for undertaking behavioral studies was based on previous studies published by our research group (84, 90, 91). Doses ranging from 100 to 3000μg/10μL were given intrathecally at 14 days prior to the tests. The animals were submitted to the assessment of nociception in formalin and OD-1 tests on day 28 after ASO delivery.

Tissue collection was performed at the end of experiments. The ASO optimal dose was chosen considering the analgesic profile observed at the behavioral tests. Next, the analgesic profile from ASO optimal dose was evaluated in carrageenan inflammatory pain model or in paw-pressure test using a Randal-Sellito apparatus (87, 96). These two models are reported to be affected in NaV 1.7 knockouts (97).

1.4. In vitro characterization of ATTO488PTx-II binding in Nav1.7 transfected HEK cells

To analyze the feasibility of the ligand binding, we initially constructed a model system using Human Embryonic Kidney (HEK) cells transfected with HaloTag-NaV 1.7 DNA sequences. In this system, the NaV 1.7 channels can be labeled using a specific TMR ligand that targets the Halo-tag expressed at NaV 1.7 proteins (98, 99). The purpose was to define the optimal concentration to label NaV 1.7 channels using the ATTO488-PTx II antagonist (488-PTx II), a fluorescent NaV 1.7 antagonist (100). Briefly, cells were plated onto glass coverslips coated with poly-D-lysine at 20-30% confluence. After 24 hours, half of the wells of a 12-well plate were transfected using 1 µg of plasmid carrying a mouse NaV 1.7-HaloTag DNA sequences, along with 20 µL of Lipofectamine 2000 (transfection reagent). The plasmid was kindly provided by Dr. Rajesh Khanna (University of Florida, Gainesville, Florida, USA). The media was replaced 4 hours after the start of the transfection. After 24 hours, cells were placed on ice and stained with a Halo ligand (Promega®, G825A, cell permeable TMR ligand; 2.5

μM final concentration) and ATTO488PTx-II (Smartox®; 0.5, 2.5 or 10 μM final concentration) for 30 minutes. After one wash in Tyrode solution (as recommend by (100) cells were fixed in 4% PFA and mounted onto microscope slides. Each concentration of ATTO488PTx-II was examined in 3 transfected samples and 1 untransfected sample (used as a control for nonspecific binding). Samples were imaged with a Leica TCS SP5 confocal system to determine the fluorescence intensity, covariance, and colocalization between the signal of two ligands. In all images obtained, the data was analyzed using the LASX Office version 1.4.7 software.

1.5. Dorsal root ganglia cell culture

Immediately after the behavioral tests, rats were euthanized under deep anesthesia using 5% Isoflurane followed by decapitation. The DRGs were harvested to perform cell culture as previously described (101) with some modifications. The Ganglia were incubated with 2% collagenase (type IV; Gibco®; catalog number 17104019) for 120 minutes and 0.25% Trypsin (Gibco®; catalog number 25200056) for 15 minutes. After two washes with DMEM (Gibco®), the cells were dissociated using glass Pasteur pipettes followed by filtration through a 70μm cell strainer for debris removal, and centrifugation at 1400 rpm for 10 minutes. The cell pellet was resuspended in DMEM and subsequently submitted to 10% bovine serum albumin gradient separation followed by centrifugation at 1400 rpm for 10 minutes. The residual debris were removed and the cells plated in 25 mm coverslips coated with poly-D-lysine/Laminin (0.02mg/mL; 0.016 mg/mL, respectively) and further disposed on 35mm single-dish cell culture plates (Thermo scientific™; catalog number 130180). All cultures were maintained in DMEM plus 1% penicillin (50 U/mL)/streptomycin (50 mg/mL), 10% Fetal bovine serum (Sigma-Aldrich; catalog number F2442), at 37 °C with 5% CO_2 until the

beginning of experiments or mRNA extraction.

1.5.1. Tissue processing for binding assays

For binding assays, the ipsilateral or contralateral L1-L5 DRGs from (n= 4-10) animals were pooled and used to obtain DRG cell cultures as previously described. The cell pellets were divided into 2 pools. The 1st pool consisted of $1x10^4$ cells (6 wells per group in each set of experiments) plated in a 96-well plate coated with poly-D-lysine/Laminin. After a 24-hour plating, these cells were lysed with RLT Buffer (QIAGEN®) and further analyzed for *Scn9a* mRNA quantification through qPCR. The 2nd pool consisted of cells plated in 25 mm poly-D-lysine/Laminin coated coverslips at 40-75% confluence. After 24-hour plating, these cells were employed in binding assays with ATTO488PTx-II and further fluorescence imaging.

1.6. Imaging experiments

1.6.1. *Assessment of ATTO488PTx-II binding in dorsal root ganglia cell cultures*

To determine the NaV 1.7 selective binding in under naïve conditions in NaV 1.7 ASO and Mismatch groups, rats were injected with the optimal dose of NaV 1.7 ASO or Mismatch and sacrificed on day 14th after ASO delivery. The L1-L5 DRGs were bilaterally harvested to obtain DRG cell cultures as previously mentioned (item 1.5). The binding for ATTO488PTx-II (488-PTxII) was assessed in 24h-plated DRG cell cultures and performed as follows: cells were washed once with Tyrode solution for 5 minutes following blockade with 2% BSA for 45 minutes and staining with 10 μM ATTO488-PTx II (diluted in Tyrode solution) for 30 minutes. Next, coverslips were fixed with 4% PFA and the slides were mounted using prolong gold (Invitrogen®) containing DAPI. All 488-PTx II binding assays were carried out on the same day, and the coverslips were maintained on

ice to minimize channel endocytosis (98). Controls consisting of cells incubated without 488-PTx II were employed to assess non-specific binding. Two sets of binding analysis were performed: **i)** different slides were analyzed and an average of 70 cells expressing fluorophore were counted in each group. The normalized cell fluorescence was calculated by the following formula: (Integrated Density of cell fluorescence) – (Average Mean of Background fluorescence intensity x Cell area in micrometers); **ii)** We determined the relative percentage of cells in these analyzed slides aiming to see the remaining fraction of cells still expressing NaV 1.7 signal according to soma size. A range between 6-11 fields were analyzed in each group and the relative percentage of NaV 1.7 positive cells were calculated by the formula: [(Number of ATTO488-PTx II positive cells ÷ Number of DAPI positive cells)] x 100. A minimum of 10 cells were required in each field, and only cells displaying clear visibility from the membrane perimeter were analyzed. The basal levels of ATTO488PTx-II fluorescence in NaV 1.7 ASO and Mismatch controls were determined using an Olympus BX51 fluorescence microscope. The coverslips were mounted in microscope slides with prolong gold containing DAPI and imaged at 63x magnification. In this set of experiments, the data was analyzed using the Image J software.

1.6.2. Assessment of ATTO488PTx-II binding in dorsal root ganglia cell cultures obtained from ASO -treated rats submitted to the formalin test

At the time frame of 24 hours after the formalin test, Lumbar (L1-L5) DRGs cell cultures (one from each anatomic side: ipsilateral or contralateral) from NaV 1.7 ASO (300 and 1000 µg/10µL doses) and Mismatch (1000 µg/10µL) groups were obtained following the protocol previously described on item 1.5. As described on item 1.5.1, the cell culture pools were plated differently for NaV 1.7 binding analysis and *Scn9a* mRNA quantification. After 24 hours, cultures corresponding to each treatment set were stained with ATTO488PTx-II and lysate for mRNA extraction as described on item 1.5.1. The intensity of fluorescence in each group was assessed in whole-cell z-stacks and assumed as indicative from amount of NaV 1.7 binding calculated as previously described on item 1.6.1.

1.6.3. Z-stacks of dorsal root ganglia cell cultures

We used a Leica TCS SP5 confocal system to determine the fluorescence intensity of ATTO488PTx-II signal in z-stacks acquired from DRGs cell cultures obtained from NaV 1.7 ASO and Mismatch groups that were harvested after the formalin test. To assess the ligand signal across the z-axis in the cells, the system was set-up to perform readings at 63x magnification, using a 0.1µm step-size and at 400Hz speed. In each slide imaged, an average of 5 fields were recorded and the intensity of ligand fluorescence measured in the max projection images of z-stacks using the Leica imaging software LASX Office version 1.4.7.

1.6.4. Assessment of ATTO488PTx-II binding in sciatic nerves obtained from animals submitted to the formalin test

At 24 hours after the formalin test, sciatic nerves from each animal of NaV 1.7 ASO (300 and 1000 µg/10µL doses) and Mismatch (1000 µg/10µL) groups were collected. The proximal section of the nerve was frozen on dry ice and further addressed for *Scn9a* mRNA quantification. The medial section of the sciatic nerve was placed on 4%PFA for fixation. After 24hs, the nerves were gradually embedded on 10-30% sucrose solution and finally placed on O.C.T compound (Tissue Tek, Sakura ™, Japan) cryo-solution to be sectioned at - 20°C in a cryostat. Sections of 20 µm from ipsilateral sciatic nerves were obtained and placed on slides (Superfrost plus; Fisherdbrand®) to be stained with ATTO488PTx-II. Slides were dried at room temperature for 30 minutes, followed by a series of washes with 0.01M PBS (pH 7.4). Unspecific binding of the secondary antibody was prevented by incubating the tissue with a blocking solution containing 3% normal donkey serum (Millipore™, S30), 0.3% Triton X-100 (Sigma, cat: 9036-19-5) in 0.01M PBS for 60 minutes. Thereafter, slides were incubated at 4°C overnight with an anti-PGP9.5 antibody (1:2000, Cedarlane, CL7756) or during 48 hours with ATTO488PTx-II. After the incubation with PGP9.5, slides were incubated with a donkey anti rabbit Cy2 antibody (1:200, Jackson ImmunoResearch, 711-005-152) for 2 hours, followed by a counterstaining with DAPI (1:1000) and coverslipped with ProLong Gold antifade Mounting (Invitrogen, P36934). Slides incubated with ATTO488PTx-II, were followed by counterstaining with DAPI and coverslipped with ProLong Gold antifade mounting. Slides were kept at room temperature for 24 hours before imaging. Z-stack images were acquired using a Zeiss LSM 800 microscope. Images were transformed into maximum projections with

ZEN software from Zeiss. A single ROI was acquired from five different sciatic nerve sections separated by 40 µm each.

1.7. RNA extraction and quantitative real-time PCR analysis

At the completion of an *in vivo* study, animals were deeply anesthetized with isoflurane and tissue harvesting was performed according to each study experimental design. For analysis of DRG neuronal cell cultures, cells were dissociated and plated. After 24 hours cells were washed three times for 5 minutes with Dulbecco's phosphate buffered saline (dPBS 1x; Gibco®) and then lysate with RLT buffer (Qiagen®) at 100 µL/well for RNA extraction following manufacturer's instruction [Qiagen, Valencia, CA]. For tissues, samples were homogenized in Trizol reagent (Thermofisher scientific, Waltham, MA) and total RNA was extracted using the Life Technologies mini-RNA purification kit (Qiagen, Valencia, CA) according to the manufacturer's protocol. After purification, the RNA samples were subjected to real-time RT-PCR analysis using the Life Technologies ABI QuantStudio 7 Flex Sequence Detection System (Applied Biosystems Inc, Carlsbad, CA). Briefly, 10 µl RT-PCR reactions containing 400 nl of RNA were run with the AgPath- ID One-Step qRT-PCR Kit (Thermofisher scientific, Waltham, MA) reagents and the primer probe sets (supplemental table-2). All qPCR reactions were run in triplicate. Outliers in triplicates due to technical issues were eliminated. Target mRNA was then normalized to *Ppia*, a ubiquitously expressed housekeeping gene, and this was further normalized to the level measured in control animals that were administered PBS or Mismatch.

1.8. Statistical analysis

The analysis of the results was performed using GraphPad Prism v.10 software (GraphPad®, San Diego, USA). The normal distribution of values in each analysis was performed with the D'Agostino Pearson, Shapiro-Wilk or Kolmorogov-Smirnov test, according to the sample size. When a non-parametric statistical test was performed, the Mann-Whitney test was used to compare two means. When the comparison involved more than two means, the Kruskal-Wallis test was performed with Dunn's post-hoc test to determine if there were differences between the groups analyzed. If the values followed a normal distribution, parametric statistical tests were done. The T-student test was selected when two means were compared. When the comparison involved more than two means, a one-way or two-way analysis of variance (ANOVA) was performed according to the experimental design. When the level of significance indicated a statistical difference between the analyzed means, the Tukey or Dunnet's test was used to compare the different analyzed groups. The level of significance adopted in each analysis of the results was $p < 0.05$.

SUPPLEMENTAL FIGURES

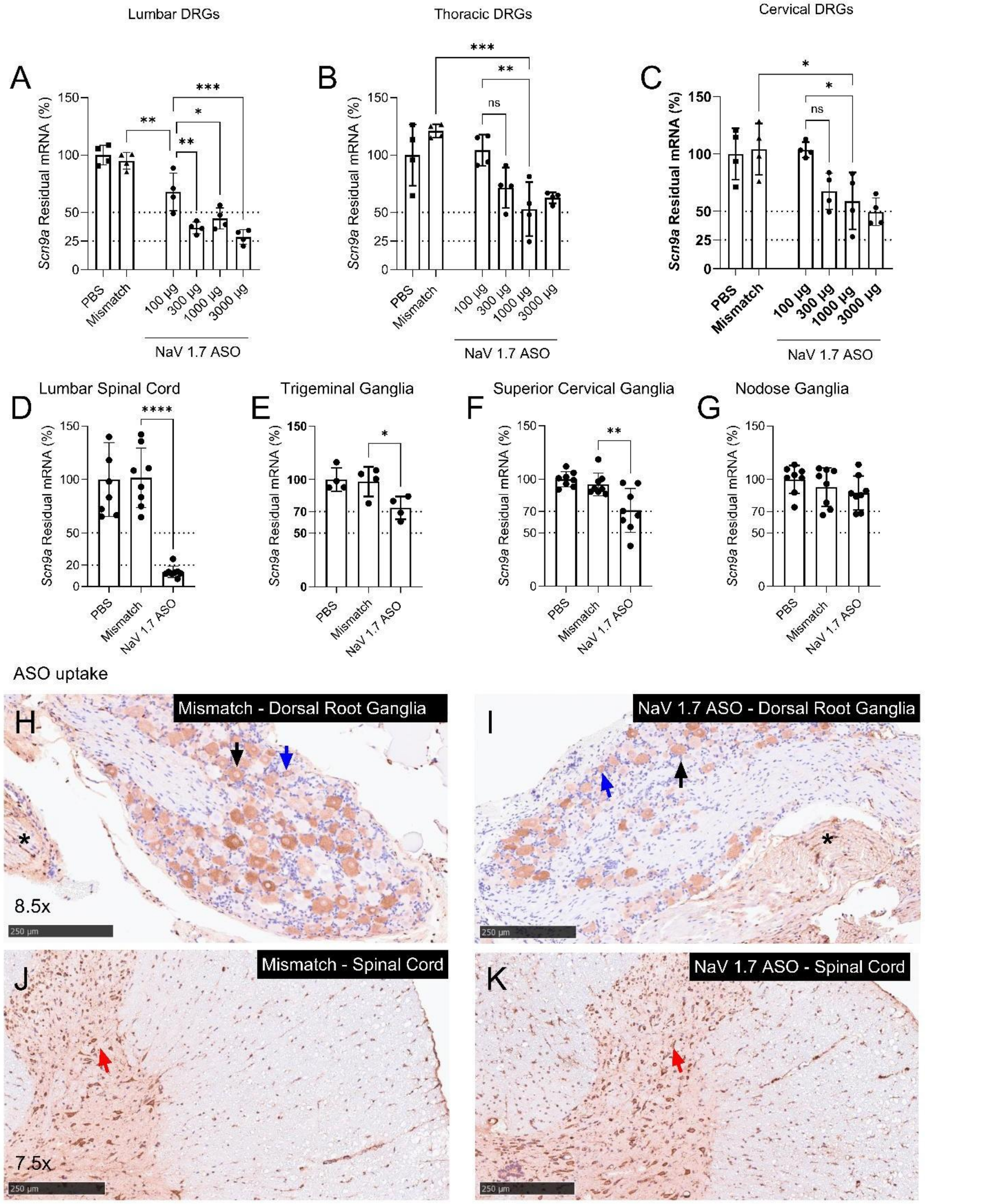

Supplemental Figure 1. Quantitative assessment of NaV 1.7 ASO knockdown on *Scn3a* mRNA and analysis oligonucleotides uptake. The intrathecal injection of NaV 1.7 ASO (100/300/1000 or 3000 µg; 10 µL) promotes a significant dose-dependent knockdown of *ScnSa* mRNA in lumbar, thoracic and cervical dorsal root ganglia in comparison with mismatch controls (**A-C**). The lumbar intrathecal delivery of NaV 1.7 antisense (NaV 1.7 ASO, 1000 µg/10 µL) induce a significant *ScnSa* mRNA knockdown locally at the lumbar section of Spinal cord (**D**), and rostrally at Trigeminal ganglia (**E**) and Superior cervical ganglia (**F**), without affecting the Scn9a mRNA levels in nodose ganglia (**G**). The lumbar intrathecal delivery of Mismatch or NaV 1.7. antisense is uptake by dorsal root ganglia spinal cord cells (**E-H**). At the DRGs, the particles of from mismatch (**H**) and NaV 1.7 ASO (**I**) oligonucleotides can be seen incorporated by

large (black arrows) and small (blue arrows) neurons. In both groups, a residual staining for antisense particles can be seen at nerve filaments projecting across the DRG (**H**, **I**, asterisks). Antisense particles could be also detected in the spinal cord parenchyma (Red arrows) of mismatch (**J**) and NaV 1.7 ASO groups (**K**). On (A-G) Results shown as mean ± standard deviation. One-way ANOVA, Tukey post-hoc test.

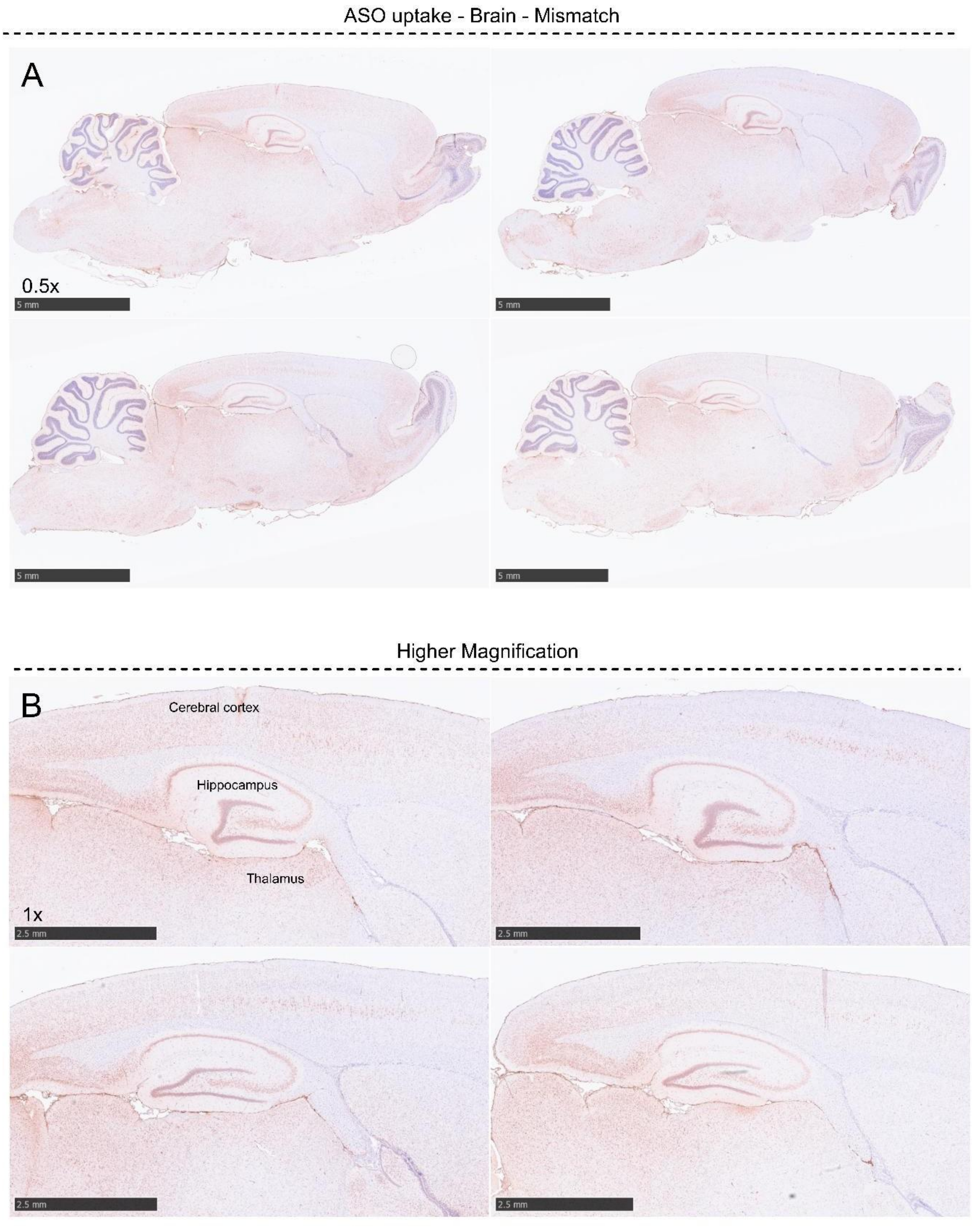

Supplemental Figure 2. Antisense oligonucleotides are uptake at the brain of mismatch animals. The intrathecal delivery of mismatch ASO leads to rostral distribution and antisense uptake at the Brain (**A**, and **B** for higher magnification). The brains were harvested 14 days after the intrathecal delivery of ASO. Each picture on panel (**A**) and (**B**) represents a different animal in which the ASO uptake was assessed. Pictures were taken at magnification of 0.5x (**A**) and 1x (**B**).

ASO uptake - Brain - NaV 1.7 ASO

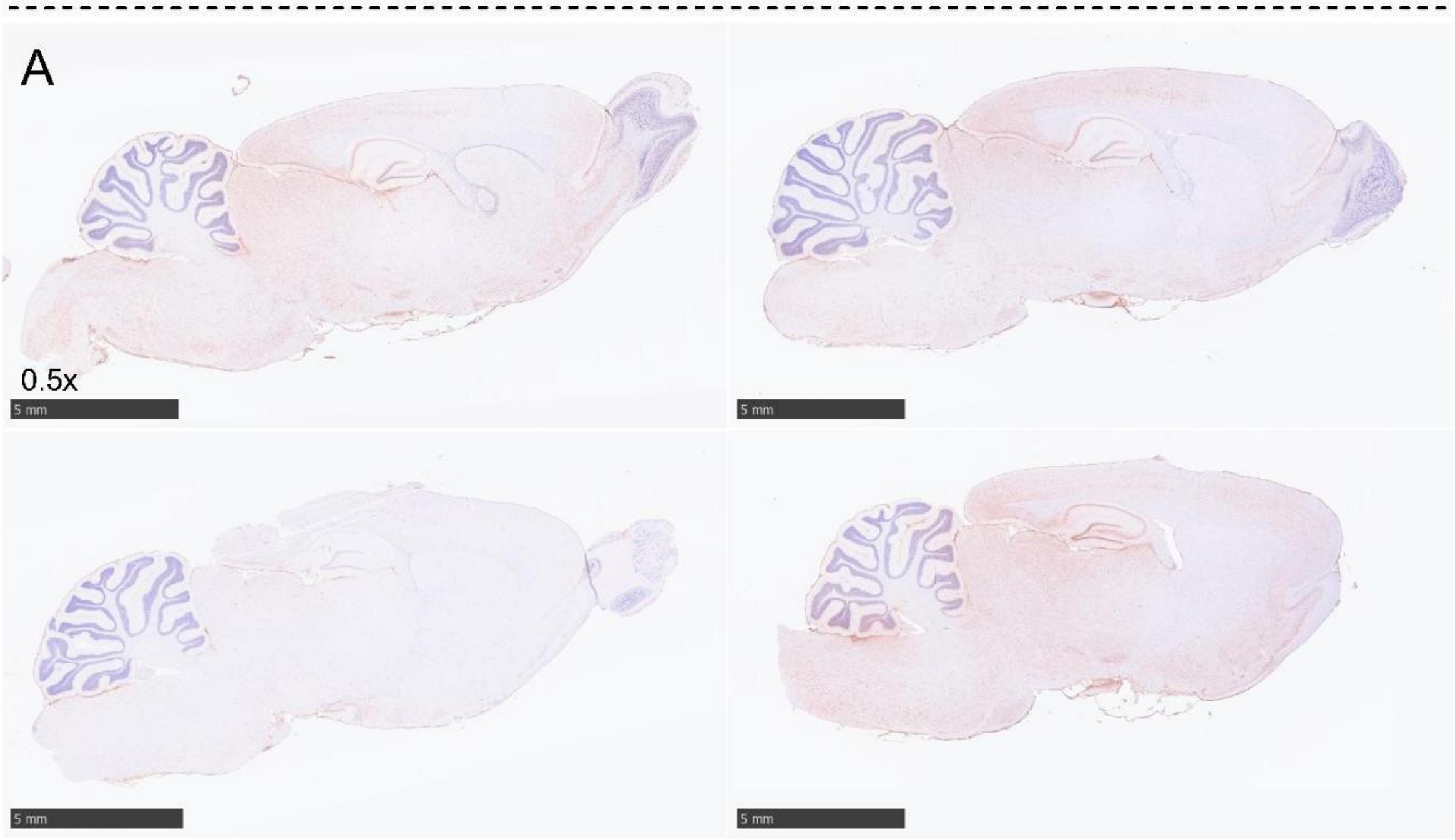

Higher Magnification

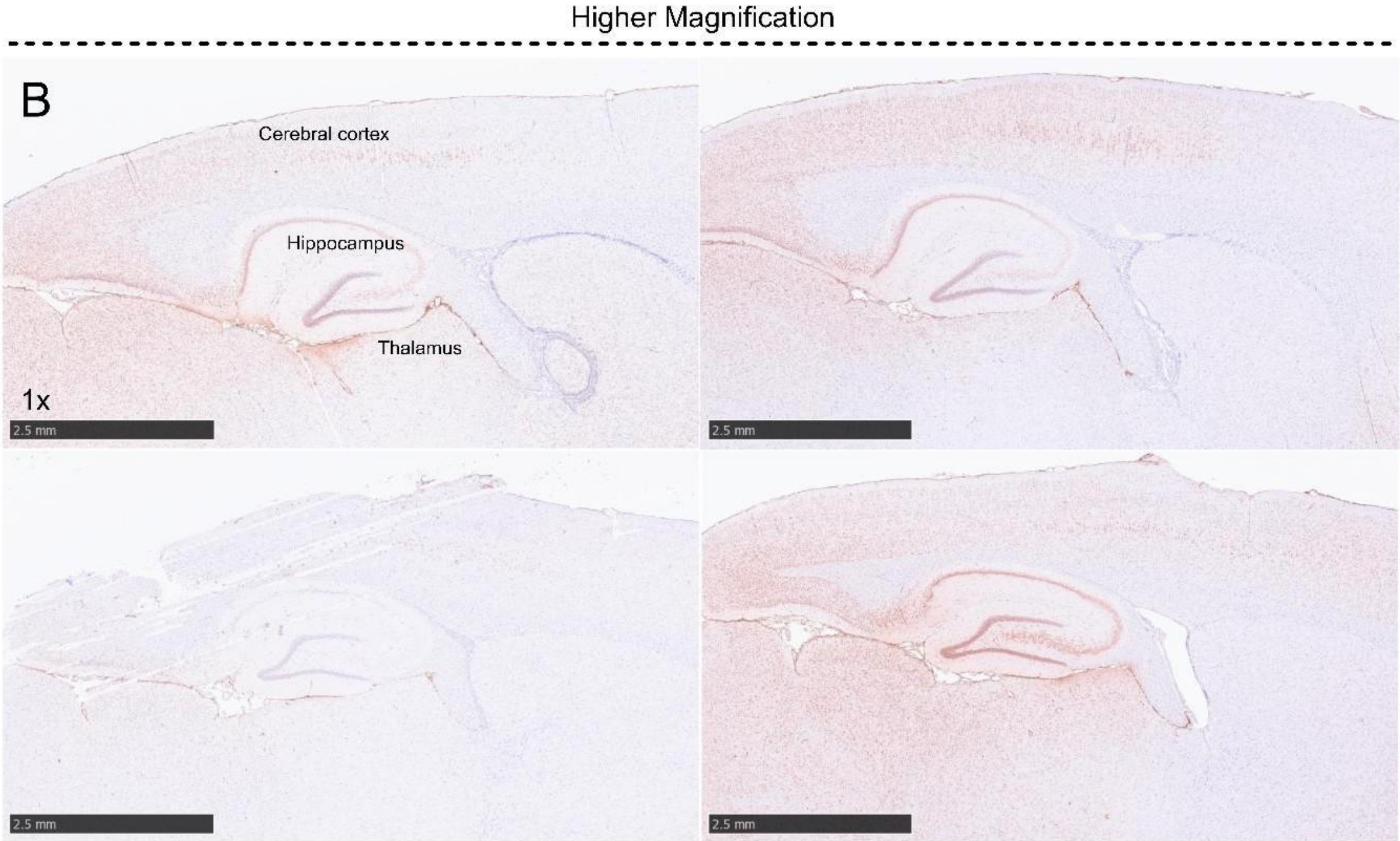

Supplemental Figure 3. Antisense oligonucleotides are uptake at the brain of NaV 1.7 ASO animals. The intrathecal delivery of NaV 1.7 ASO leads to rostral distribution and antisense uptake at the Brain (**A**, and **B** for higher magnification). The brains were harvested 14 days after the intrathecal delivery of ASO. Each picture on panel (**A**) and (**B**) represents a different animal in which the ASO uptake was assessed. Pictures were taken at magnification of 0.5x (**A**) and 1x (**B**).

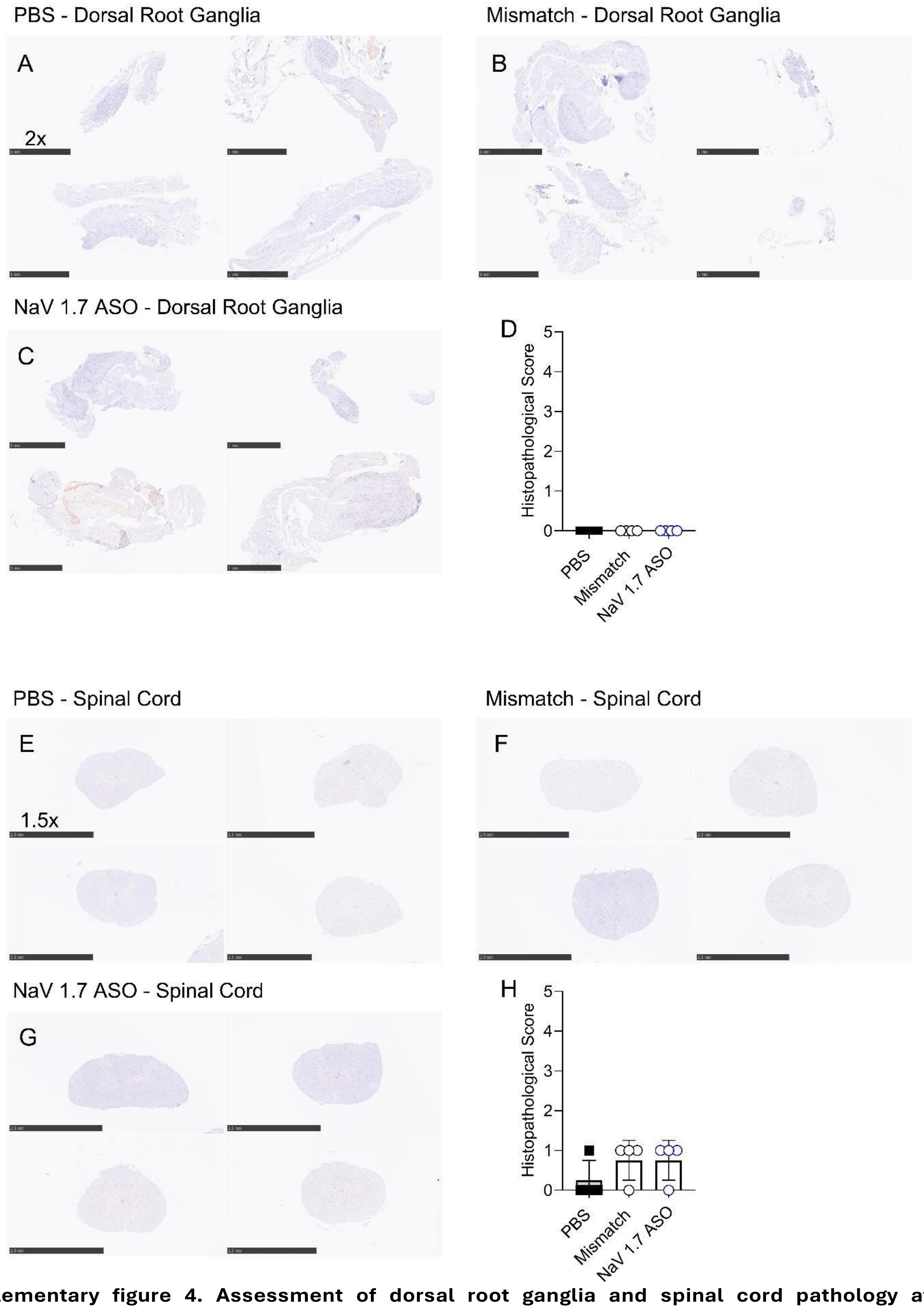

Supplementary figure 4. Assessment of dorsal root ganglia and spinal cord pathology after intrathecal antisense delivery. The intrathecal injection of Mismatch or NaV 1.7 ASO optimal dose (1000 µg; 10 µL) does not lead to significant microgliosis (IBA-1 staining) at the dorsal root ganglia (A-D) or spinal cord (E-H) in comparison with PBS controls. Each picture on panel (**A**, **B**, **C**) and (**E**, **F**, **G**) represents a different animal in which the pathology score was assessed. Pictures were taken at magnification of 2x (**A**, **B**, **C**) and 1.5x (**E**, **F**, **G**). On (**D/H**) Results shown as mean ± standard deviation. Kruskal-Wallis test with Dunn's post-hoc test.

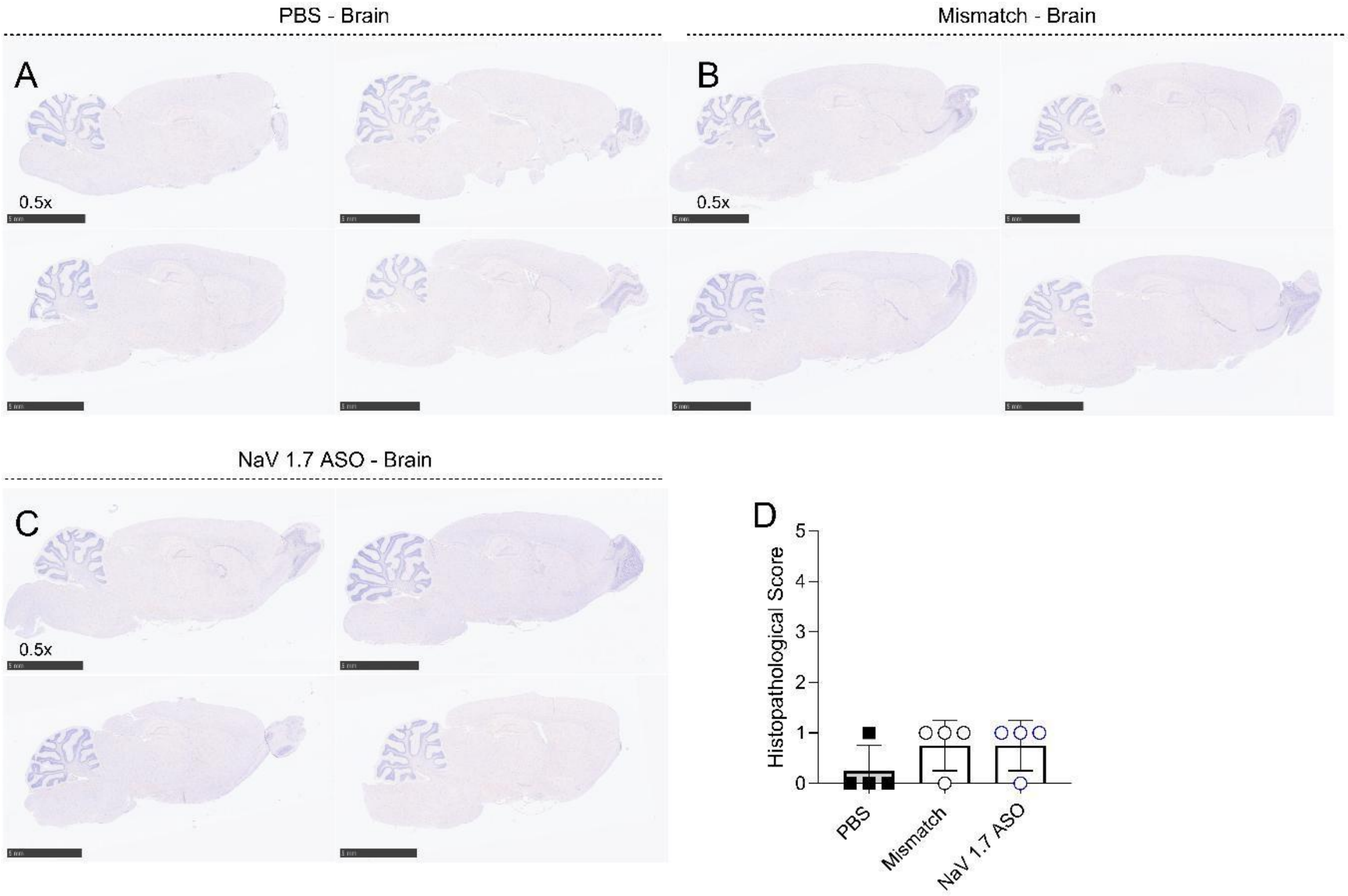

Supplementary figure 5. Assessment of Brain pathology after intrathecal antisense delivery. The intrathecal injection of Mismatch or NaV 1.7 ASO optimal dose (1000 µg; 10 µL) does not lead to significant microgliosis (IBA-1 staining) at the Brain in comparison with PBS controls. Each picture on panel (**A**, **B**, **C**) represents a different animal in which the pathology score was assessed. Pictures were taken at magnification of 0.5x. On (**D**) Results shown as mean ± standard deviation. Kruskal-Wallis test with Dunn's post-hoc test.

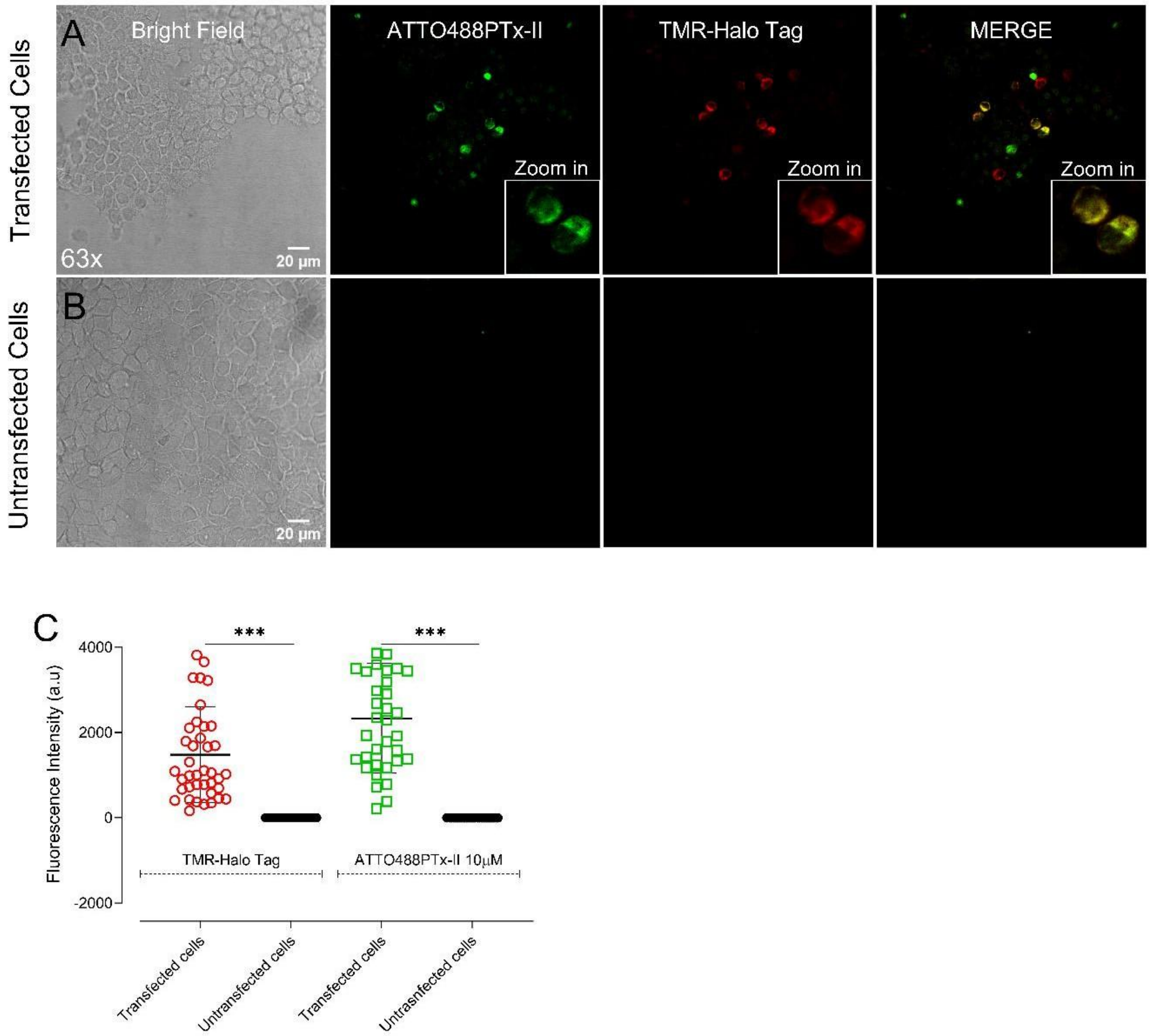

Supplemental Figure 6. HEK cells expressing NaV 1.7 channels significantly showed fluorescent signal in the presence of TMR-Ligand at 2.5 µM, and 488-PTx II at 10 µM dose. (A) HEK cells transfected with NaV 1.7 channel exhibited fluorescence signal in the presence of 488-PTx II and TMR-Ligand, indicating a selective binding from the channel antagonist which was confirmed by the colocalization at the Merge field. **(B)** No signal was detected in un-transfected cells for 488-PTx II and TMR-Ligand suggesting minimal or none off-target molecular effects. **(C)** The fluorescence intensity of transfected cells exposed to 488-PTx II and TMR-Ligand is significantly different in comparison with un-transfected cells. Results shown as mean ± standard deviation. Symbol *** indicates $P<0.001$ for comparison between groups. Welch's T-test**.**

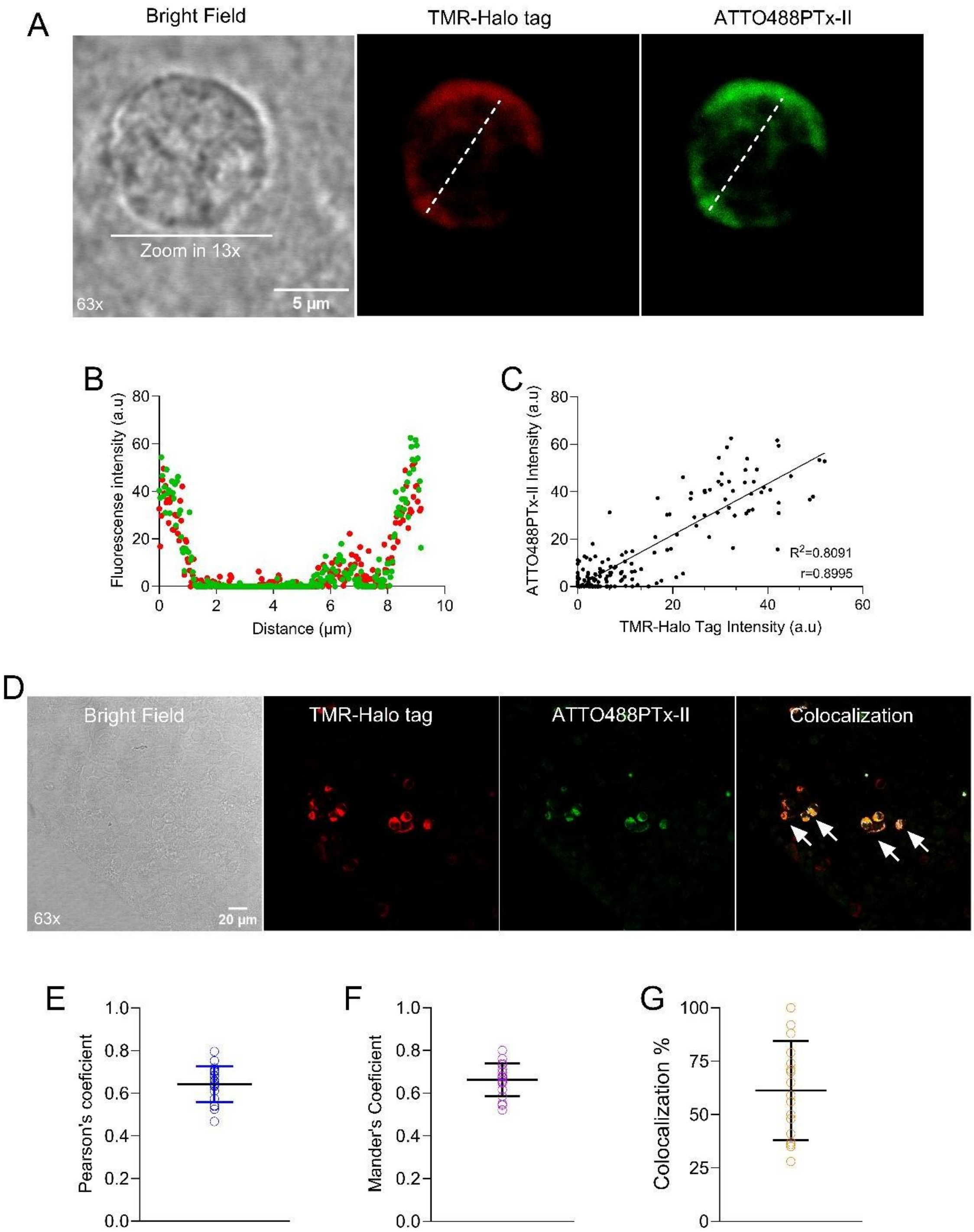

Supplemental Figure 7. The ATTO488PTx-II signals colocalizes with TMR-ligand dye indicating a selective binding with the NaV 1.7 antagonist. **(A) and (B)** Signal covariance analysis performed showing the fluorescent profile plot from the cell transfected with NaV 1.7 plasmid and stained with TMR-Ligand + 488-PTxII**.** The corresponding correlation analysis between the signal intensity for the two ligands is shown in **(C)**. The HEK cells expressing NaV 1.7 exhibited colocalized dots shared between the stained sites for 488-PTx II and TMR ligand (**D**) with an average of 60% colocalization **(G)**. Overall, both Pearson's and Mander's correlations in all samples analyzed highlighted the colocalization data showing an average correlation **(E)** and an overlapping coefficient **(F)** around 0.6. In **(B)** results are shown as the mean fluorescence intensity detected for both ligands (Y axis) across the distance analyzed (x axis). In **(C)** results shown as the mean fluorescence intensity detected (Y axis ATTO488-PTx II; X axis TMR-ligand for Halo tag) for the ligands. In **(E)**, **(F)**, and **(G)** the results are present as mean ± standard deviation and shows, respectively, the data acquired for pearson's and mander's coefficient, and colocalization.

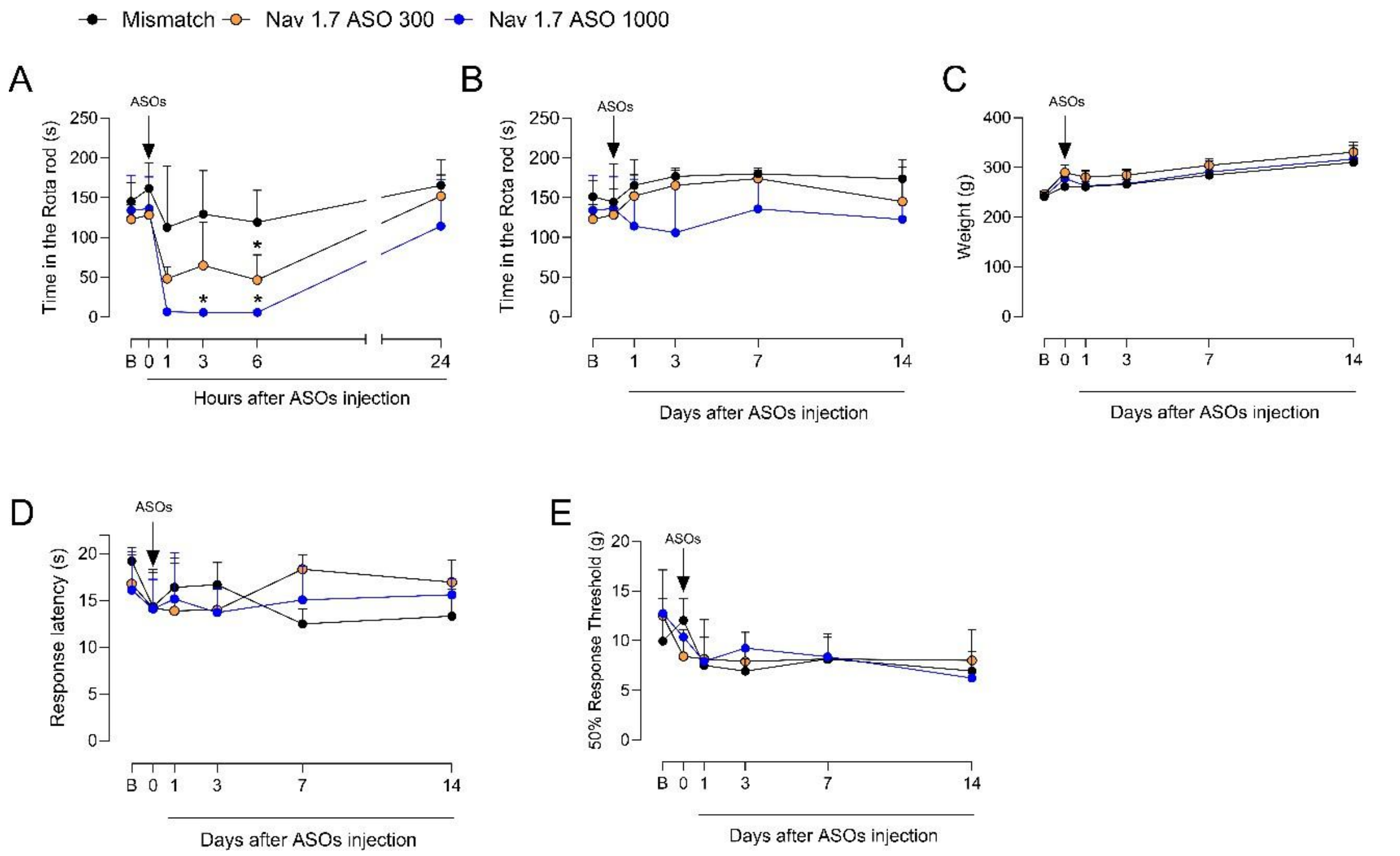

Supplementary Figure 8. Assessment of motor function, tactile and thermal thresholds from different doses of ASO. The intrathecal injection of ASO (300 µg/10µL or 1000 µg/10µL) led to a significant decrease in rota-rod activity at 3, 6h after ASO delivery **(A)**. This impairment on motor function is not seen day 1- day14 after ASOs injection **(B)**, where animals also show normal weight-gain **(C).** No changes in thermal (**D**) or tactile (**E**) thresholds were detected between the 2 doses tested in comparison with mismatch controls. Results shown as mean ± standard deviation. Symbol * indicate P<0.05 in comparison with mismatch controls. From A-E, Two-way ANOVA with Tukey post-hoc test.

SUPPLEMENTAL TABLES

**ASO**	**Full Sequence**
Control	**CsCoToAoTsAsGsGsAsCsTsAsTsCsCsAoGoGoAsA**
Rat Scn9a	**GsCoCoAoToAsAsTsCsCsGsGsGsTsTsToCoTsGsC**
	**Legends:**
	2'-O-Methoxyethylribose
	Phosphodiester
	Deoxyribose
	Phosphorothioate

Supplemental Table-1. ASO sequence and chemistry

Gene	Protein Target	Forward	Reverse
Scn1a	NaV 1.1	GGATGAGGGTGGTTGTGAAT	ACGCCCATGATGCTGAAA
Scn2a	NaV 1.2	CATTCAACACGCACATGGATTC	TGCCATCTTTTCATCCTGCT
Scn8a	NaV 1.6	CCGAGAGCAAGCTCAAGAAA	CCCGTAGATGAAAGGCAAACT
Scn9a	NaV 1.7	CAGAGGAAAGCATCCGAAAGA	CAGGGACCCGCGAATG
Scn10a	NaV 1.8	TGCGGGCTACTTGAACGAA	TCAAGCTCCTCAATGACAGAAGTC
Scn11a	NaV 1.9	AGTCTCGCTGATGTGCTCTA	CAGGATTTGGCTAACTTGAAGAC
Aif-1	IBA-1	AGGAGAAAAACAAAGAACACCAGAA	CAATTAGGGCAACTCAGAAATAGCT
Gfap	GFAP	GAAACCAGCCTGGACACCAA	TCCACAGTCTTTACCACGATGTTC
Ppia	Cyclophilin	CCCACCGTGTTCTTCGACA	AAACAGCTCGAAGCAGACGC

Supplemental Table-2. Primers used in this study

Score 1	< 10% microglia cells are activated in a specific area
Score 2	30% microglia cells are activated in a specific area
Score 3	30 - 50% microglia cells are activated in a specific area
Score 4	50 - 75% microglia cells are activated in a specific area
Score 5	> 75% microglia cells are activated in a specific area

Supplemental Table-3. Score system used to assess microgliosis across dorsal root ganglia, spinal cord, and brain.

SUPPLEMENTAL REFERENCES

83. Malkmus SA, Yaksh TL. Intrathecal Catheterization and Drug Delivery in the Rat. Pain Research. Totowa, NJ: Humana Press; 2004:109–121.

84. Mazur C, et al. Development of a simple, rapid, and robust intrathecal catheterization method in the rat. J Neurosci Methods. 2017;280:36–46.

85. Yaksh TL, Rudy TA. Analgesia Mediated by a Direct Spinal Action of Narcotics. Science (1979). 1976;192(4246):1357–1358.

86. Chaplan SR, et al. Quantitative assessment of tactile allodynia in the rat paw. J Neurosci Methods. 1994;53(1):55–63.

87. RANDALL LO, SELITTO JJ. A method for measurement of analgesic activity on inflamed tissue. Arch Int Pharmacodyn Ther. 1957;111(4):409–19. 88. Dirig DM, et al. Characterization of variables defining hindpaw withdrawal latency evoked by radiant thermal stimuli. 1997.

89. Yaksh TL, et al. An automated flinch detecting system for use in the formalin nociceptive bioassay. 2001.

90. Fitzsimmons BL, et al. Role of spinal p38α and β MAPK in inflammatory hyperalgesia and spinal COX-2 expression. Neuroreport. 2010;21(4):313–317.

91. Mohan A, et al. Antisense oligonucleotides selectively suppress target RNA in nociceptive neurons of the pain system and can ameliorate mechanical pain. Pain. 2018;159(1):139–149.

92. Bennett DL, et al. The Role of Voltage-Gated Sodium Channels in Pain Signaling. Physiol Rev. 2019;99(2):1079–1151.

93. Ho C, O'Leary ME. Single-cell analysis of sodium channel expression in dorsal root ganglion neurons. Molecular and Cellular Neuroscience. 2011;46(1):159–166.

94. Iseppon F, et al. Sodium channels Nav1.7, Nav1.8 and pain; two distinct mechanisms for Nav1.7 null analgesia. Neurobiology of Pain. 2024;16:100168.

95. Takeuchi H. Neurotoxicity by microglia: Mechanisms and potential therapeutic strategy. Clin Exp Neuroimmunol. 2010;1(1):12–21.

96. Deuis JR, Dvorakova LS, Vetter I. Methods used to evaluate pain behaviors in rodents [preprint]. Front Mol Neurosci. 2017;10. https://doi.org/10.3389/fnmol.2017.00284.

97. Minett MS, et al. Distinct Nav1.7-dependent pain sensations require different sets of sensory and sympathetic neurons. Nat Commun. 2012;3(1):791.

98. Higerd-Rusli GP, et al. The fates of internalized NaV1.7 channels in sensory neurons: Retrograde cotransport with other ion channels, axon-specific recycling, and degradation. Journal of Biological Chemistry. 2023;299(1). https://doi.org/10.1016/j.jbc.2022.102816.

99. Los G V., Wood K. The HaloTagTM: A Novel Technology for Cell Imaging and Protein Analysis. High Content Screening. New Jersey: Humana Press; :195–208.

100. Montnach J, et al. Fluorescent- and tagged-protoxin II peptides: potent markers of the Nav1.7 channel pain target. Br J Pharmacol. 2021;178(13):2632–2650.

101. Lemes JBP, et al. Participation of satellite glial cells of the dorsal root ganglia in acute nociception. Neurosci Lett. 2018;676:8–12.
